# Fiber-Reinforced Polymer Composites: Manufacturing, Properties, and Applications

**DOI:** 10.3390/polym11101667

**Published:** 2019-10-12

**Authors:** Dipen Kumar Rajak, Durgesh D. Pagar, Pradeep L. Menezes, Emanoil Linul

**Affiliations:** 1 Department of Mechanical Engineering, Sandip Institute of Technology & Research Centre, Nashik 422212, India; 2 Department of Mining Machinery Engineering, Indian Institute of Technology (ISM), Dhanbad 826004, India; 3 Department of Mechanical Engineering, K. K. Wagh Institute of Engineering Education & Research, Nashik 422003, India; durgeshpagar90@gmail.com; 4 Department of Mechanical Engineering, University of Nevada, Reno, NV 89557, USA; pmenezes@unr.edu; 5 Department of Mechanics and Strength of Materials, Politehnica University of Timisoara, 300 222 Timisoara, Romania; 6 National Institute of Research for Electrochemistry and Condensed Matter, 300 569 Timisoara, Romania

**Keywords:** fiber-reinforced polymer, composite materials, natural fibers, synthetic fibers

## Abstract

Composites have been found to be the most promising and discerning material available in this century. Presently, composites reinforced with fibers of synthetic or natural materials are gaining more importance as demands for lightweight materials with high strength for specific applications are growing in the market. Fiber-reinforced polymer composite offers not only high strength to weight ratio, but also reveals exceptional properties such as high durability; stiffness; damping property; flexural strength; and resistance to corrosion, wear, impact, and fire. These wide ranges of diverse features have led composite materials to find applications in mechanical, construction, aerospace, automobile, biomedical, marine, and many other manufacturing industries. Performance of composite materials predominantly depends on their constituent elements and manufacturing techniques, therefore, functional properties of various fibers available worldwide, their classifications, and the manufacturing techniques used to fabricate the composite materials need to be studied in order to figure out the optimized characteristic of the material for the desired application. An overview of a diverse range of fibers, their properties, functionality, classification, and various fiber composite manufacturing techniques is presented to discover the optimized fiber-reinforced composite material for significant applications. Their exceptional performance in the numerous fields of applications have made fiber-reinforced composite materials a promising alternative over solitary metals or alloys.

## 1. Introduction

Rapid growth in manufacturing industries has led to the need for the betterment of materials in terms of strength, stiffness, density, and lower cost with improved sustainability. Composite materials have emerged as one of the materials possessing such betterment in properties serving their potential in a variety of applications [1,2,3,4]. Composite materials are an amalgamation of two or more constituents, one of which is present in the matrix phase, and another one could be in particle or fiber form. The utilization of natural or synthetic fibers in the fabrication of composite materials has revealed significant applications in a variety of fields such as construction, mechanical, automobile, aerospace, biomedical, and marine [5,6,7,8].

Research studies from the past two decades have presented composites as an alternative over many conventional materials as there is a significant enhancement in the structural, mechanical, and tribological properties of fiber-reinforced composite (FRC) material [9,10,11]. Though composite materials succeeded in increasing the durability of the material, currently a strong concern regarding the accumulation of plastic waste in the environment has arisen [12]. This concern has compelled researchers around the world to develop environmentally friendly materials associated with cleaner manufacturing processes [13,14,15]. Several different composite recycling processes also have been developed to cope with the thousands of tons of composite waste generated in a year. Mechanical recycling includes pulverization, where decreased sized recyclates are being used as filler materials for sheet molding compounds. In thermal recycling, degradation of composite waste by pyrolysis is done or an enormous amount of heat energy is obtained by burning composite materials with a high calorific value. There also exist more efficient processes such as chemical recycling (solvolysis) and high-voltage fragmentation (HVF). The addition of natural fillers such as natural fibers, cellulose nanocrystals, and nanofibrillated cellulose in the polymers matrix to fabricate eco-friendly composites has improved material properties while minimizing the problem regarding residue accumulation [16,17,18,19].

Many researchers have reported advantages of cellulosic fibers, such as being abundantly available in nature, nontoxic, renewable, cost-effective, and also providing necessary bonding with the cement-based matrix for significant enhancements in properties such as ductility, toughness, flexural capacity, and impact resistance of a material [20,21,22]. In modern techniques, inclusion of fly ash, limestone powder, brick powder, and many other mineral additives are used to strengthen the composite structures. Fracture toughness has been enhanced with the addition of fly ash in a concrete composite for structural applications resulting in increased lifespan of the material [23,24]. Natural fibers are mainly classified as fibers that are plant-based, animal-based, and mineral-based. As the asbestos content in the mineral-based fibers is hazardous to human health, these are not well-explored fibers with respect to research into fiber-reinforced composite materials, while plant-based fibers provide promising characteristics such as lower cost, biodegradable nature, availability, and good physical and mechanical properties [25,26]. Plant fibers include leaf fibers (sisal and abaca), bast fibers (flax, jute, hemp, ramie, and kenaf), grass and reed fibers (rice husk), core fibers (hemp, jute, and kenaf), seed fibers (cotton, kapok, and coir), and all other types, which may include wood and roots. Polymer matrices are also divided into a natural matrix and a synthetic matrix, which is petrochemical-based and includes polyester, polypropylene (PP), polyethylene (PE), and epoxy [27].

The latest research contributes the development of hybrid composites with the combination of natural and synthetic fibers. The composite structures consisting of more than one type of fiber are defined as hybrid composites. There are methods to combine these fibers, which involve stacking layers of fibers, the intermingling of fibers, mixing two types of fibers in the same layer making interplay hybrid, selective placement of fiber where it is needed for better force, and placing each fiber according to specific orientation [28]. Among all these, stacking of fibers is the easiest procedure, and others introduce some complications in obtaining a positive hybridization effect. Many researchers got success by developing optimized composite materials for efficient use in particular applications by varying fiber content, its orientation, size, or manufacturing processes. It is necessary to understand the physical, mechanical, electrical, and thermal properties of FRCs for their effective application. FRCs are currently being employed in copious fields of applications due to their significant mechanical properties. These composite materials sometimes depart from their designed specifications as some defects, such as manufacturing defects, cause them to deviate from the expected enhancement in mechanical properties. These manufacturing defects involve misalignment, waviness, and sometimes breakage of fibers, fiber/matrix debonding, delamination, and formation of voids in the matrix of a composite material. An increase of 1% voids content in composites and leads to a decrease in tensile strength (10–20%), flexural strength (10%), and interlaminar shear strength (5–10%), respectively. It can be eradicated by manipulating the processing parameters of manufacturing processes [29].

Therefore, there is a need to understand and study different types of composite manufacturing techniques to implement optimized techniques that will avoid defects and give apposite self-sustaining, durable composite material that is efficient for the desired field of application. There are many conventional manufacturing techniques for fabrication of a composite material that have been in practice for the past few decades and some of the recently developed automated composite manufacturing techniques use robot assistance for processing, which leads to complete automation and an immense rise in productivity [30].

## 2. Classification

**Figure 1:**
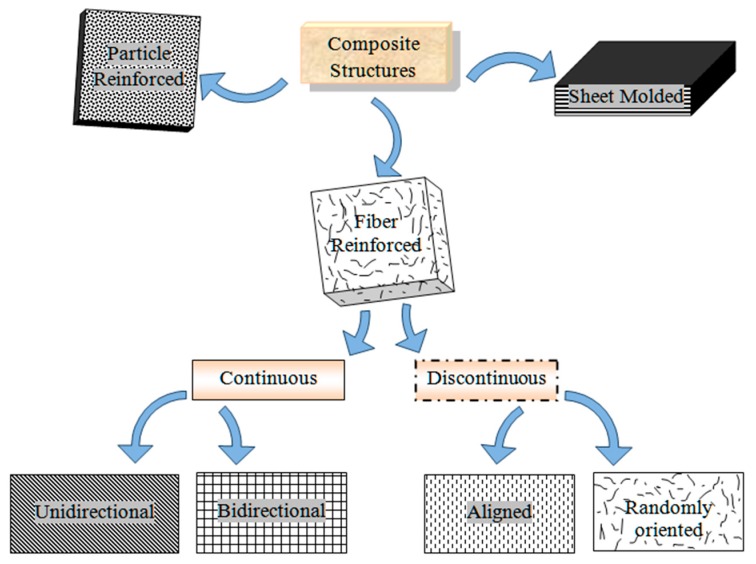
Classification of composites.

Composite materials are classified according to their content, i.e., base material and filler material. The base material, which binds or holds the filler material in structures, is termed as a matrix or a binder material, while filler material is present in the form of sheets, fragments, particles, fibers, or whiskers of natural or synthetic material. As represented in Figure 1, composites are classified into three main categories based on their structure [31].

### 2.1. Fiber-Reinforced Composites

Composites consist of fibers in the matrix structure and can be classified according to fiber length. Composites with long fiber reinforcements are termed as continuous fiber reinforcement composites, while composites with short fiber reinforcements are termed as discontinuous fiber reinforcement composites. Hybrid fiber-reinforced composites are those where two or more types of fibers are reinforced in a single matrix structure [32]. Fibers can be placed unidirectionally or bidirectionally in the matrix structure of continuous fiber composites, and they take loads from the matrix to the fiber in a very easy and effective way. Discontinuous fibers must have sufficient length for effective load transfer and to restrain the growth of cracks from avoiding material failure in the case of brittle matrices. The arrangement and orientation of fibers define the properties and structural behavior of composite material [33,34]. Improvement in properties such as impact toughness and fatigue strength can be seen with the use of chemically treated natural fibers. Fibers of glass, carbon, basalt, and aramid in the dispersed phase were conventionally used in the matrix structure of a fiber-reinforced polymer (FRP) composite materials [35,36]. Significant properties of natural fiber polymer composites (NFPCs) have potential applications in the modern industry, as researchers currently are compelled towards the development of environmentally friendly materials due to stringent environmental laws.

**Figure 2:**
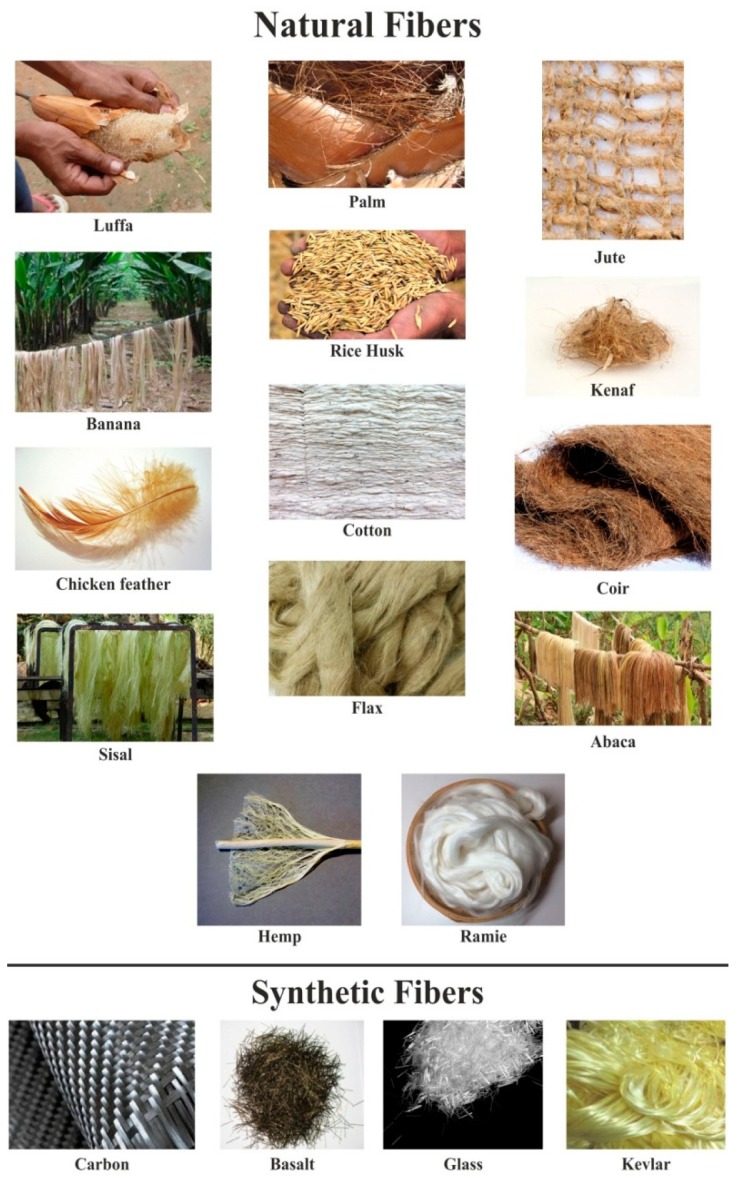
Classification of fibers, reproduced from [37,38,39,40,41,42,43,44,45,46,47,48,49,50,51,52,53] under open access license.

There are numerous fibers available for composite materials and they are primarily categorized as natural or synthetic fibers. Further, recent studies have revealed unprecedented material properties when these two fibers are combined together, blending with a matrix material to form a hybrid composite. Some of the natural and synthetic fibers are shown in Figure 2.

#### 2.1.1. Synthetic Fibers

Human-made fibers that are produced by chemical synthesis are called synthetic fibers and further classified as organic or inorganic based on their content [54]. Generally, the strength and stiffness of fiber materials are much higher than that of the matrix material, making them a load-bearing element in the composite structure [55,56,57,58,59].

Glass fibers (GFs) are most widely used among all the synthetic fibers as they offer excellent strength and durability, thermal stability, resistance to impact, chemical, friction, and wear properties. However, the machining of glass fiber-reinforced polymers (GFRPs) is relatively slow, challenging, and shows reduced tool life while working on conventional machining systems [60]. GFs also carry the disadvantage of disposal at the end of their service life [61].

However in some applications, more stiffness is required, so carbon fibers (CFs) are employed instead of GFs. Although some of the other types of synthetic fibers like aramid, basalt, polyacrylonitrile (PAN-F), polyethylene terephthalate (PET-F), or polypropylene fibers (PP-F) offer some advantages, they are rarely used in thermoplastic short-fiber-reinforced polymers (SFRP); they have been used for specific applications where their desired properties are applicable [62]. Carbon fiber-reinforced polymer (CFRP) composites have revealed numerous applications in aerospace, automobile, sports, and many other industries [63,64,65]. Young’s modulus of solids and foams increased by 78% and 113%, respectively, when the weight percentage of carbon fibers increased from 10% to 30%. The improvement in the cellular structure resulted in the improvement of Young’s modulus of the foams by 35% when carbon fiber/polypropylene (CF/PP) was used to make composite foams prepared by microcellular injection molding [66].

Graphene fibers are a new type of high-performance carbonaceous fibers that reflect high tensile strength with enhanced electrical conductivity when compared to carbon fibers. Several enhanced properties of graphene fibers show their potentiality in a variety of applications, such as lightweight conductive cables and wires, knittable supercapacitors, micromotors, solar cell textiles, actuators, etc. [67,68]. The molecular dynamics simulation of polymer composites with graphene reinforcements showed increases in Young’s modulus, shear modulus, and hardness by 150%, 27.6%, and 35%, respectively. Furthermore, a reduction in the coefficient of friction and abrasion rate by 35% and 48% was achieved [69].

Basalt fiber (BF) possesses better physical and mechanical properties over fiberglass. In addition, BF is significantly cheaper than carbon fibers. The effect of temperature on basalt fiber-reinforced polymer (BFRP) composites has been investigated, where there was an increase in static strength and fatigue life at a certain maximum stress observed with a decrease in temperatures [70].

Thermal properties of Kevlar fiber-reinforced composites (KFRCs) are enhanced by hybridizing it with glass or carbon fibers, though there is less research on the hybridization of Kevlar fibers (KFs) with natural fibers. KFRCs show high impact strength with a high degree of tensile properties, but due to their anisotropic nature they possesses low compression strength compared to their glass and carbon fiber counterparts [71].

#### 2.1.2. Natural Fibers

Natural fibers (NFs) are a very easy to obtain, extensively available material in nature. They reveal some outstanding material properties like biodegradability, low cost per unit volume, high strength, and specific stiffness. Composites made of NF reinforcements seem to carry some diverse properties over synthetic fibers, such as reduced weight, cost, toxicity, environmental pollution, and recyclability. These economic and environmental benefits of NF composites make them predominant over synthetic fiber-reinforced composites for modern applications [33]. Depending on the type, natural fibers have similar structures with different compositions. The inclusion of long and short natural fibers in thermoset matrices has manifested high-performance applications [72,73].

Sisal fiber (SF)-based composites are frequently being used for automobile interiors and upholstery in furniture due to their good tribological properties. When SFs were reinforced with polyester composites, the tensile strength increased with fiber volume and when reinforced with polyethylene (PE) composites, tensile strength of 12.5 MPa was observed in 6 mm long sisal fibers [74,75,76].

Hemp composite showed a 52% increase in specific flexural strength of a material when compared to GF-reinforced composite with a propylene matrix [77]. Composite material with 5% maleic anhydride-grafted polypropylene (MAPP) by weight mixed with polypropylene (PP) matrix that was reinforced with 15%, by weight, alkaline-treated hemp fibers manifested advancement in flexural and tensile strength by 37% and 68%, respectively [78].

Polylactic acid (PLA) thermoplastic composites with kenaf fiber reinforcement possess tensile and flexural strength of 223 MPa and 254 MPa, respectively [79]. Also, before laminating, removing absorbed water from the fibers results in the improvement of both flexural and tensile properties of kenaf fiber laminates [80,81]. Previously, polyester samples without any reinforcements showed flexural strength and flexural modulus of 42.24 MPa and 3.61 GPa respectively, while after reinforcement of 11.1% alkali-treated virgin kenaf fibers in unsaturated polyester matrix, composite material showed flexural strength and flexural modulus of 69.5 MPa and 7.11 GPa [82].

The sound and vibration behavior of flax fiber-reinforced polypropylene composites (FF/PPs) have been investigated using a sound transmission loss (STL) test. The results showed an increase in stiffness, damping ratio, and mass per unit area of the material due to increase in transmission loss, as the material possesses high sound absorption properties [83,84]. Use of short flax fiber (FF) laminates resulted in an enhancement in tensile properties of a material. Also, the material strength and shear modulus increased by 15% and 46%, respectively, with 45° fiber orientation [85].

The study on the free vibration characteristics of ramie fiber-reinforced polypropylene composites (RF/PPs) showed that higher fiber content in a polymer matrix leads to slippage between the fiber and the matrix, and this leads to an increase in the damping ratio during the flexural vibration. That means that an increase in fiber content results in enhancement in damping properties of RF/PP composite [86,87].

During the growth of a rice grain, a natural sheath forms around the grain, known as a rice husk (RH), which is treated as agricultural waste, but it is utilized as reinforcement in composite materials to investigate enhancement in material properties [88,89]. For the enhancement of the acoustic characteristics of the material, 5% of RH in polyurethane (PU) foam displayed optimum sound absorption performance [90].

Composite material consisting of 5% chicken feathers as reinforcement fibers with epoxy resin as matrix material showed optimum results following an impact test. Moreover, these chicken feathers used with 1% of carbon residuum (CR) fused with epoxy resin formed a hybrid composite, which displayed substantial enhancement in tensile, flexural, and impact strength of a material [91].

It has been seen that along with the length of a raw jute reed, tensile strength and bundle strength decrease from root to tip, with the root portion-based composite carrying 44% and 35% higher tensile and flexural strength, respectively, than that of the composites made from the tip portion of raw jute reed [92,93].

Randomly oriented coir fiber-reinforced polypropylene composites offers higher damping properties than synthetic fiber-reinforced composites. High resin content offers higher damping properties, therefore, lower fiber loading leads to more energy absorption. The maximum damping ratio of 0.4736 was obtained at 10% of fiber content in coir–PP composite, while further increasing fiber content to 30% showed improved natural frequency of material to 20.92 Hz [94,95].

Palm fibers (PFs) showed outstanding fiber-matrix interfacial interaction. Also, the addition of palm fibers in low-density polyethylene (LDPE) resulted in higher Young’s modulus compared to homo-polymers [96].

Friction composites are fabricated using abaca fiber (AF) reinforcement, which offers excellent wear resistance property with a wear rate of 2.864 × 10^−7^ cm^3^/Nm at 3% of fiber content. Also, the density decreased with increasing abaca fiber content [97].

The addition of luffa fibers (LFs) as a reinforcement constituent of composite material resulted in the advancement of the mechanical properties like tensile, compressive, flexural, impact strength, and water absorption characteristics of a material [98]. Adding a 9.6 wt % of LFs in epoxy matrix displayed a decrement in the density of the material by 3.12%, which further resulted in the reduction in material weight [99].

Energy absorption and load-carrying capacities of a tube material have been improved with the implementation of cotton fiber epoxy composite [100]. Manufacturing techniques and applications of some fibers with their matrix materials are depicted in Table 1.

#### 2.1.3. Hybrid Fibers

Thermoplastic composites reinforced with natural fiber, in general, show poor strength performance when compared to thermoset composites. Therefore, to acquire benefits of design flexibility and recycling possibilities, these natural fiber composites are hybridized with small amounts of synthetic fibers to make them more desirable for technical applications. Hemp/glass fiber hybrid polypropylene composites exhibited flexural strength of 101 MPa and 5.5 GPa flexural modulus when filler content of 25% hemp and 15% glass was present in a composite structure by weight. An enhancement in impact strength and water absorption properties of the material was also perceived [101]. A scanning electron microscopy (SEM) study revealed excellent interfacial bonding between the fiber and the matrix of oil palm/kenaf fiber-reinforced epoxy hybrid composite that evince the improvement in the tensile and flexural properties of the material. Moreover, when compared to other composites, oil palm/kenaf fiber hybrid composite absorbs more energy during impact loading that makes the hybrid material a good competitor in the automotive sector [102]. A hybrid composite comprised of carbon and flax fibers reinforcement in the matrix of epoxy resin resulted in 17.98% reduction in the average weight of the material, and maximum interlaminar shear strength (ILSS) of 4.9 MPa and hardness of 77.66 HRC was observed [103]. Fiber hybridization is a promising strategy, where two or more types of fibers are combined in a matrix of composite material to mitigate the drawback of the type of fiber, keeping benefits of others. Synergetic effects of both the fibers aids to enhance properties of the composite material that neither of the constituents owned [104,105]. A hybrid composite made of epoxy resin as matrix material that had a reinforcement of 27% banana along with 9% jute fibers showed a tensile strength of 29.467 MPa. Another composite with the same matrix material that had reinforcement of 21.5% coconut sheath and 15.5% jute fibers showed a compressive strength of 33.87 MPa. An increasing amount of banana fiber reinforcements resulted in increased tensile strength of the composite material [106].

### 2.2. Particle-Reinforced Composite

Compared to FRC, particle-reinforced composite (PRC) is not that effective by means of material strength and fracture resistance property. However, ceramic, metal, or inorganic particles restrict the deformation and provide good material stiffness. In recent days, PRCs are also getting a bit of attention due to their isotropic properties and cost-effectiveness. Moreover, these composites are manufactured using similar techniques used for monolithic material [107,108]. PRCs are employed for civil applications such as roadways and concrete structures, where a high degree of wear resistance is expected. In concrete, cement acts as a binder material while aggregate of coarse rock or gravel as a filler material provides hardness and stiffness [109].

### 2.3. Sheet-Molded Composites

Sheet-molded composites (SMCs) are fabricated by bonding homogeneous layers of materials using a compression molding process to form nonhomogeneous composite laminates. The laminate is composed of layers and, in the case of FRP composites made of fiber sheets, buckling stability of the material improves with increasing the number of layers in the laminate [110]. SMC shows the application in large structural components like automotive body parts consisting of high strength to weight ratio [111,112,113]. Tensile properties of natural fibers can be defined by their chemical compositions. Tensile strength increases with an increase in cellulose content of the fibers, and decreases with increase in lignin content. Some of the properties of frequently used fibers are displayed in Table 2, and Table 3 depicted different properties offered by matrix material.

There are several factors, other than composite constituents and manufacturing processes, that influence the FRP composite performance.

Interphase: It is the region around the fiber in a matrix phase of a FRP composite structure. At the interphase stress, transfer from matrix to fiber takes place at loading conditions. Therefore, to evaluate the performance of composite, not only the properties of its constituent materials, but also understanding the behavior of interphase, is important [33].

Pretreatments: Physical or chemical treatments like preheating, alkalization, acetylation, and use of silane coupling agent on fibers to modify the fiber surface and its internal structure results in the improvement of adhesion at the interface and amalgamation of the matrix resin into the fibers [118].

Size effect: For FRP-confined cylindrical concrete columns, size effect depends on the mode of failure; there is no occurrence of size effect if failure is plasticity dominated. When failure is fracture-dominated, it occurs due to shear banding. While in large columns, cylinders of small size fail due to FRP rupture caused by plastic dilation in the concrete [119].

Confinement methods: FRP-confined high strength concrete (HSC), and ultra-high-strength concrete (UHSC) show highly ductile compressive behavior when sufficiently confined. On the other hand, if HSC or UHSC are inadequately confined, there is degradation of the axial compressive performance of the FRP tube-encased or FRP-wrapped specimen. FRP thickness and confinement method does not make much difference in the strain reduction factor (kε), while for the concrete structures, kε decreases with an increase in concrete compressive strength [120].

Cross-section: Under concentric compression, the behavior of concrete-filled fiber-reinforced polymer tubes (CFFT) depends upon amount and type of tube material used, concrete strength, cross-sectional shape, specimen size, and manufacture method. When cross-sectional shape is taken into consideration, newly developed rectangular and square CFFT shows highly ductile behavior as a significant improvement with internal FRP reinforcement, when compared to conventional CFFTs [121]. Further studies have shown that specimen size does not influence the compressive behavior of CFFTs. Although a significant correlation has been observed between fiber elastic modulus and the strain reduction factor, fibers with a higher modulus of elasticity result in a decrease of the strain factor that further resembles concrete brittleness while manufacturing CFFTs [122].

Fiber volume: Maleic anhydride-grafted polypropylene (MA-g-PP) was used as a compatibilizer to improve adhesion between bamboo fiber and polypropylene matrix composite material. Composite with 5% MA-g-PP concentration and 50% fiber volume has increased impact strength by 37%, flexural strength by 81%, flexural modulus by 150%, tensile strength by 105%, and tensile modulus by 191%. When the fiber volume of chemically treated composite with MA-g-PP compatibilizer increased from 30% to 50%, it showed an increase in the heat deflection temperature (HDT) by 23 °C to 38 °C compared to virgin PP. Therefore, fiber volume of 50% fraction, 1–6 mm fiber length with 90–125 µm fiber diameters, coupled with MA-g-PP compatibilizer is the recommended optimized composition for bamboo fiber-reinforced polypropylene composites, which results in a maximum enhancement in the mechanical properties and a higher thermal stability is also achieved [123].

Fiber orientation: When CO_2_ laser engraving was employed for material removal of GFRP, it was found that surface roughness and machined depth of the laser-engraved surface were hugely dependent upon the fiber direction [60]. T300 carbon fibers and 7901 epoxy resin as a matrix material were used to fabricate T300/7901 unidirectional (UD) fiber-reinforced composite to investigate mechanical properties in uniaxial tension/compression and torsional deformations. Micrographs of fiber matrix interface at different load levels were examined, which revealed that matrix plastic deformation has no significant effect on predicted ultimate load at failure. It also revealed a noticeable drop in the ultimate strength with the increase in fiber angle from 0° to 15°. Stress concentration factor (SCF) plays an important role while considering the failure prediction, without consideration of SCFs transverse strength will be overestimated [124]. Thermal buckling load for curvilinear fiber-reinforced composite laminates is more for antisymmetric laminates, while laminates with nonuniform temperature distribution exhibit high critical load carrying capacity [110].

## 3. Manufacturing Techniques

Manufacturing of FRP composite involves manufacturing of fiber preforms and then reinforcing these fibers with the matrix material by various techniques. Fiber preforms involve weaving, knitting, braiding, and stitching of fibers in long sheets or mat structure [125,126,127]. Preforms are used to achieve a high level of automation with the assistance of robotics, which offers control over the fiber angle and the fiber content on every zone of the part to be molded [128].

### 3.1. Conventional Manufacturing Processes

Prepregs are a combination of fibers and uncured resin, which are pre-impregnated with thermoplastic or a thermoset resin material that only needs the temperature to be activated. These prepregs are ready-to-use materials where the readily impregnated layers are cut and laid down into the open mold [128]. Dow Automotive Systems has developed VORAFUSE, a technique that combines epoxy resin with carbon fiber for prepreg applications to improve material handling and cycle time in the compression molding of composite structures. Working in collaboration with a variety of automotive companies, they have achieved significant weight reduction, which results in efficient manufacturing of CFRP composite structures [129].

**Figure 3:**
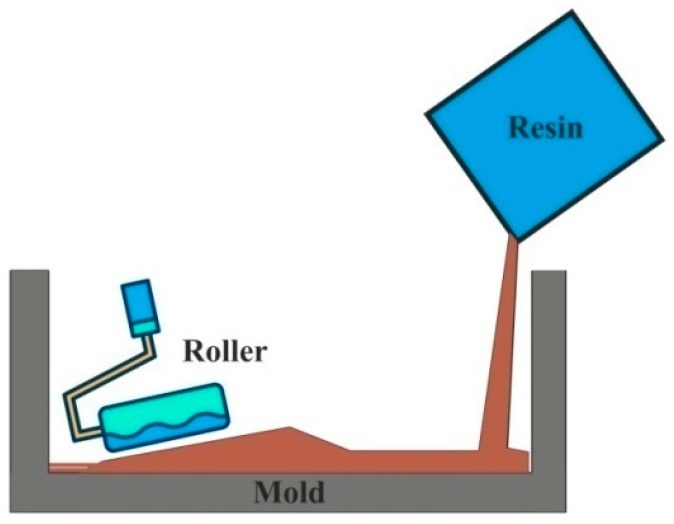
Hand layup process.

Figure 3 shows the hand lay-up, which is the most common and widely used open mold composite manufacturing process. Initially, fiber preforms are placed in a mold where a thin layer of antiadhesive coat is applied for easy extraction. The resin material is poured or applied using a brush on a reinforcement material. The roller is used to force the resin into the fabrics to ensure an enhanced interaction between the successive layers of the reinforcement and the matrix materials [130,131,132].

**Figure 4:**
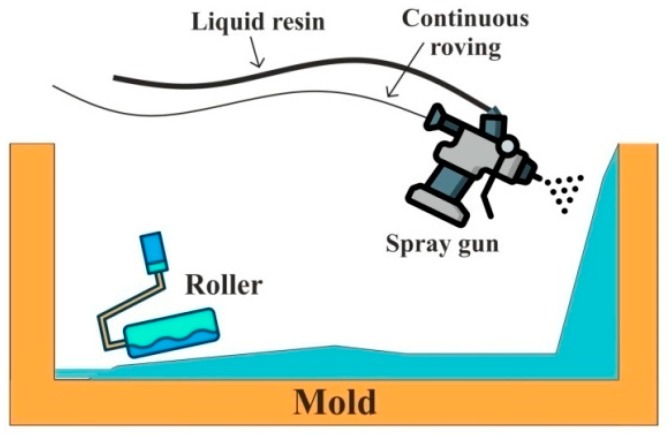
Spray-up process.

Spray-up technique is no different than hand lay-up. However, it uses a handgun that sprays resin and chopped fibers on a mold. Simultaneously, a roller is used to fuse these fibers into the matrix material. The process is illustrated in Figure 4. It is an open mold type of technique, where chopped fibers provide good conformability and quiet faster than hand lay-up [133,134].

**Figure 5:**
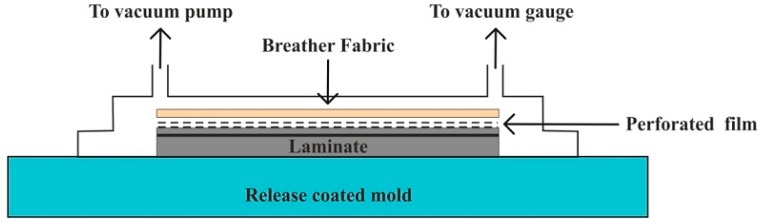
Vacuum bag molding process.

Vacuum bag molding uses a flexible film made of a material such as nylon polyethylene or polyvinyl alcohol (PVA) to enclose and seal the part from the outside air. Many times, the vacuum bag molding technique is performed with the assistance of the hand lay-up technique. Laminate is first made by using the hand lay-up technique, and then after it is placed between the vacuum bag and the mold to ensure fair infusion of fibers into the matrix material [135,136]. The air between the mold and the vacuum bag is then drawn out by a vacuum pump while atmospheric pressure compresses the part. The process can be well understood by Figure 5. Hierarchical composites were prepared with multiscale reinforcements of carbon fibers using a vacuum bagging process, which eliminated chances of detectable porosity and improper impregnation of dual reinforcements, with increases in flexural and interlaminar shear properties by 15% and 18%, respectively [137].

**Figure 6:**
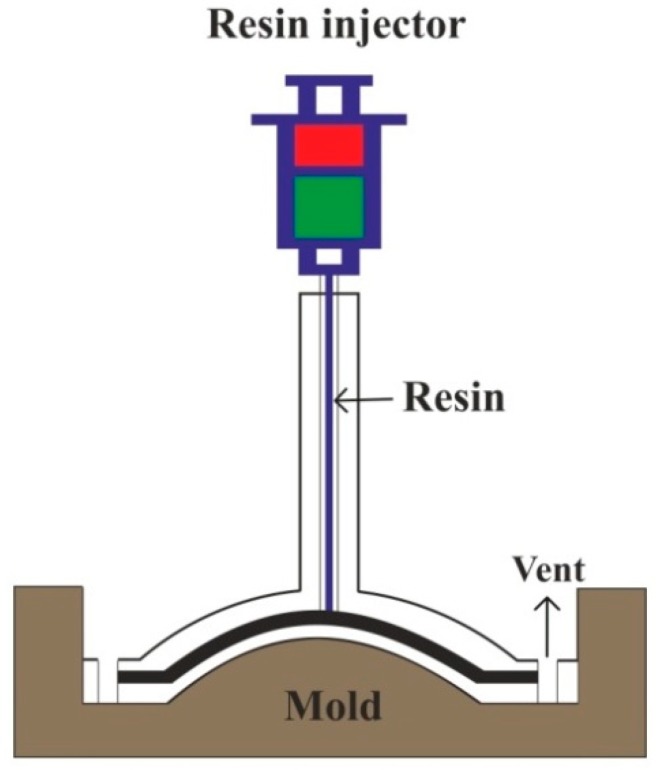
Resin transfer molding process.

The preform fiber reinforcement mat or woven roving arranged at the bottom half of the mold and preheated resin is pumped under pressure through an injector [132]. The mechanism of the resin transfer molding (RTM) process can be understood with Figure 6. A variety of combinations of fiber material with its orientation, including 3D reinforcements, can be achieved by RTM [138,139]. It produces high-quality, high-strength composite structural parts with surface quality matching to the surface of the mold [140].

**Figure 7:**
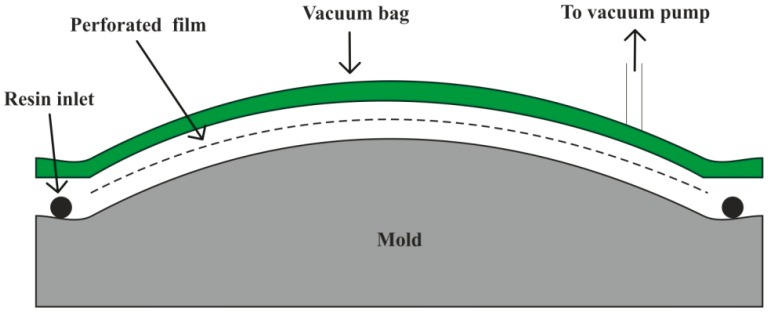
Vacuum infusion process.

Vacuum infusion or vacuum assisted resin transfer molding (VARTM) is a recent development, in which preform fibers are placed on a mold and a perforated tube is positioned between vacuum bag and resin container. Vacuum force causes the resin to be sucked through the perforated tubes over the fibers to consolidate the laminate structure, as shown in Figure 7. This process leaves no room for excess air in the composite structure, making it popular for manufacturing large objects like boat hulls and wind turbine blades [141,142]. For the improvement in the strength of textile composites, natural fibers are surface treated. Alkali treated flax fiber-reinforced epoxy acrylate resin composite fabricated using VARTM technique resulted in improvement of tensile strength by 19.7% [143].

**Figure 8:**
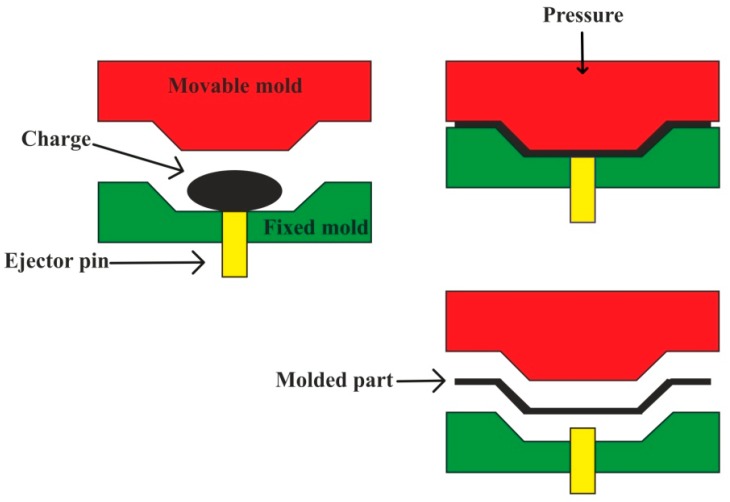
Compression molding process.

It uses preheated molds mounted on a hydraulic or mechanical press. A prepared reinforcement package from prepreg is placed in between the two halves of the mold, which are then pressed against each other to get a desired shape of the mold. Figure 8 represents the stepwise processing of compression molding. It offers short cycle time, a high degree of productivity, and automation with dimensional stability, hence it finds diverse applications in the automobile industry [144,145,146]. Dispersion of 35% filler elements containing sisal fiber and zirconium dioxide (ZrO_2_) particles in the matrix of unsaturated polyester (UP) was obtained by the compression molding technique, which displayed optimum mechanical properties when tested under SEM, X-ray diffraction, and Fourier transform infrared spectrometer (FTIR) [147]. Jute fiber-reinforced epoxy polymer matrix-based composite has been fabricated by using hand lay-up followed by the compression molding technique at s curing temperature ranging from 80 °C to 130 °C. Enhancement in the mechanical properties has been observed with the maximum tensile strength of 32.3 MPa, flexural strength of 41.8 MPa, and impact strength of 3.5 Joules [148].

**Figure 9:**
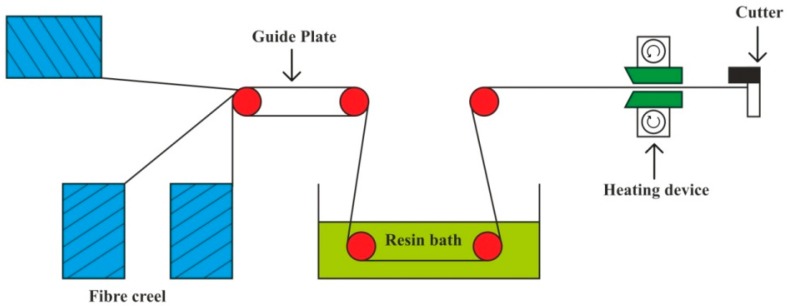
Pultrusion process.

The pultrusion process can be explained (Figure 9) as strands of continuous fibers are pulled through a resin bath, which are further consolidated in a heated die. It is a continuous process, useful for fabrication of composites with a constant cross-section with a relatively longer length; it enables production with a high degree of automation and lower production cost [149,150,151].

**Figure 10:**
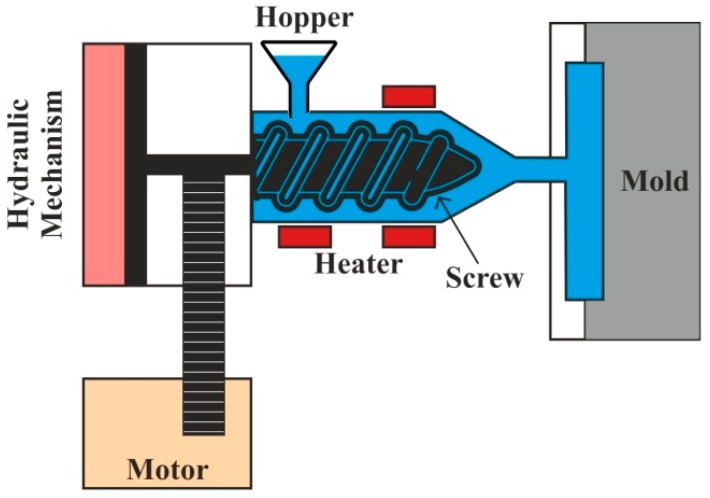
Injection molding process.

Injection molding has the ability to fabricate composite parts with high precision and at very low cycle times. In a typical injection molding process, fiber composites in the form of pellets are fed through a hopper, and then they are conveyed by a screw with a heated barrel, as shown in Figure 10. Once the required amount of material is melted in a barrel, the screw injects the material through a nozzle into the mold. where it is cooled and acquires the desired shape [152]. Injection molding is found to be very effective for thermoplastic encapsulations of electronic products required in medical industries [153]. Improvement in fiber-matrix compatibility and uniformity in the dispersion of fibers in the matrix material is achieved during the surface treatments of biocomposites [154].

### 3.2. Advance Manufacturing Processes

**Figure 11:**
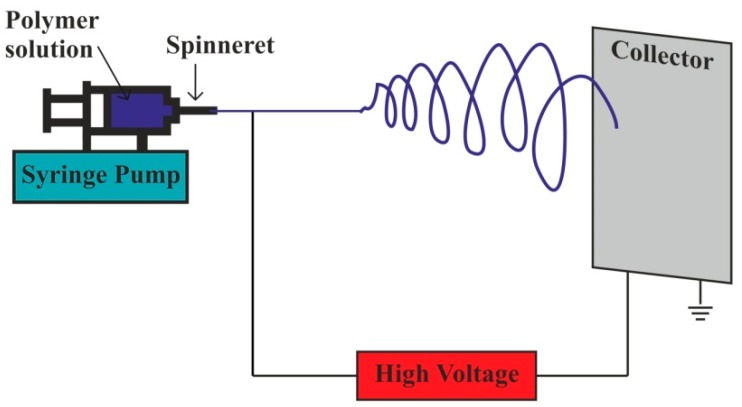
Electrospinning process.

The emerging nanotechnology has provoked researchers to seek out new nanoscale fiber manufacturing techniques for composite manufacturing. An electrostatic fiber fabrication technique called electrospinning uses electrical forces to generate continuous fibers of two nanometers to several micrometers. Polymer solution ejected through spinneret forms a continuous fiber, which is collected at the collector shown in Figure 11. It serves enhanced physical and mechanical properties, flexibility over process parameters, high surface area to volume ratio, and high porosity; therefore it finds potential in diverse fields of biomedical applications such as wound healing, tissue engineering scaffolds, drug delivery, as a membrane in biosensors, immobilization of enzymes, cosmetics, etc. [155,156].

Additive manufacturing (AM) offers a high level of geometrical complexity for the fabrication of fully customized objects as it takes advantage of computer-aided designing and also eliminates the requirement of molds, which saves cost and time of manufacturing process [157,158]. AM is one of the leading technologies in composite manufacturing as it provides wide range over the selection of fiber volume and fiber orientation. It has the ability to transverse design idea into the final product quickly without the wasting material and cycle time, which makes it ideal for prototyping and individualization [159,160,161].

Specially developed manufacturing techniques: The fabrication of carbon fiber-reinforced metal matrix composites (CF-MMC) involves powder metallurgy, diffusion bonding, melt stirring, squeeze casting, liquid infiltration, ion plating, and plasma spraying. Each one of them serves distinct benefits for manufacturing CF-MMC. Powder metallurgy and melt stirring being simplest and most economical; diffusion bonding uses specially designed tools, where carbon fiber preforms are prepared by infiltration in polymer binder and then stacked up with metal sheets. Slurry casting is carried out at the freezing temperature of the metal matrix material, eliminating the probability of interfacial reactions and degradations of the interface [162].

### 3.3. Automated Manufacturing Techniques

**Figure 12:**
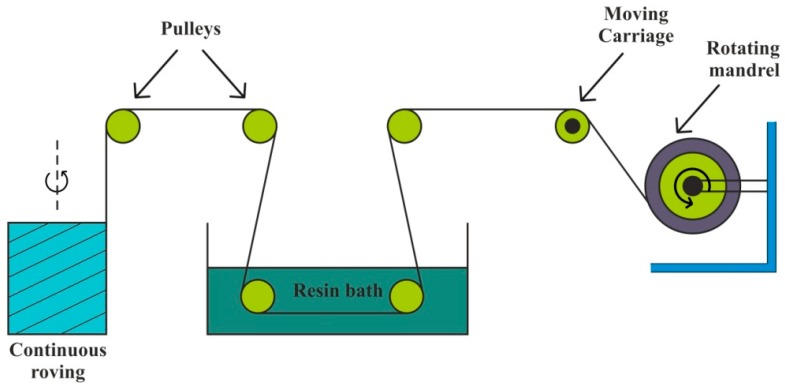
Filament winding.

It is a continuous process that offers self-automation, which leads to reduced cost. Filament winding is useful to create axisymmetric, as well as some non-axisymmetric, composite parts, such as pipe bends [163]. Driven by several pulleys, continuous prepreg sheets, rovings, and monofilament are made to pass through a resin bath and collected over a rotating mandrel, as displayed in Figure 12. Then, after applying sufficient layers, mandrel, which has the desired shape of the product, is set for curing at the room temperature [164,165]. Recently developed robotic filament winding (RFW) technique is provided with an industrial robot equipped with a feed and deposition system. It yields advantages over process control, repeatability, and manufacturing time by replacing a human operator [166].

Automated tape layup (ATL) and automated fiber placement (AFP) techniques are efficient for large, flat, or single curvature composite structures as it uses the assistance of a multiaxis articulating robot, where the material is deposited in accordance with a defined computer numerical control (CNC) path. The AFP process involves the individual prepreg lay-up of laminates onto a mandrel using a numerically controlled fiber placement machine, which are then further pulled off by holding spools [167]. Composite structures are fabricated quickly and accurately, but the expenses in employing required specialized equipment keep these technologies out of reach for small to medium scale manufacturers [168].

## 4. Applications

### 4.1. Civil

Fire resistant concrete: For many years, FRP composites have been widely used to strengthen the concrete structures and recent studies have introduced inorganic/cementitious materials to develop fiber-reinforced inorganic polymer (FRiP) composites. Phosphate cement-based FRiP is used to replace the epoxy in the FRP composite structure with improvement in fire resistance [169,170,171,172,173]. These inorganic cementitious materials consist of Portland cement, phosphate-based cement, alkali-activated cement, or magnesium oxy-chloride cement (MOC). FRiP retains about 47% of it strengthening efficiency when exposed to fire [174,175,176]. FRP sandwich material is a special form of laminated composite material, which offers high strength to weight ratio, thermal insulation, and service life benefits. Therefore, it has emerged as an excellent alternative to metallic skins for sandwich composites in structural engineering applications. Also, FRP sandwich systems provide more durable and cost-effective infrastructure in bridge beams, footbridges and bridge decks, multifunctional roofs, cladding and roofing systems for buildings, railway sleepers, and floating and protective structures [177].

**Figure 13:**
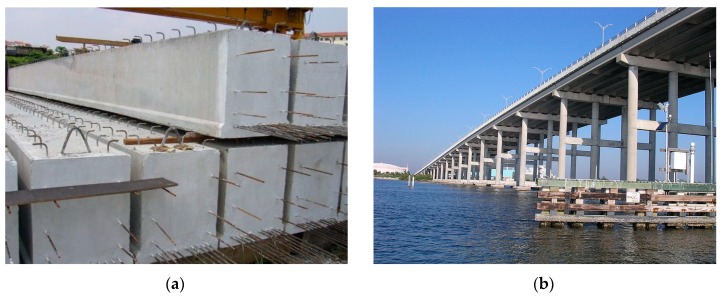
Reinforced composite (RC) beams (**a**), concrete bridge (**b**), reproduced from [184,185] under open access license.

Concrete beams: A significant improvement in flexural strength and load-carrying capacity is observed in FRP sheets bonded to the tension face of concrete beams, even when subjected to the harsh environment of wet and dry cycling [178]. To achieve higher means of strain levels, the anchorage of externally bonded FRP materials is applied prior to the premature debonding failure of reinforced concrete (RC) structures. Among the rest of the anchorage solutions, FRP anchors were found to be 46% more effective than vertically orientated U-jacket anchors, resulting in remarkably high anchorage efficiency. Simplicity, non-destructiveness, and ease of application are some other advantages for FRP to concrete applications [179]. The newly developed basalt microfibers are added longitudinally as reinforcement to the concrete structures to study its feasibility and flexural behavior; it exhibits improvement in curvature ductility with increased maximum moment capacity of the beams. Regardless of the type of concrete used, there is an enhancement in the flexural capacity of the beams with an increase in BFRP reinforcement ratio [180,181,182]. Figure 13a shows some RC beams. RC members can be strengthened by employing FRP anchors with varying fiber content and embedment angle to enhance the strain capacity of externally bonded FRP composites. As the anchor dowel angle increases relative to the direction of load, there is an increase in the strength of the joint with a decrease in ductility of joint [183].

Bridge system: For applications such as constructing durable concrete structures and restoring aged structures like bridges and tunnels, sprayable ultra-high toughness cementitious composite (UHTCC) is implemented. The UHTCC improved the durability of concrete structures with higher compressive, tensile, and flexural strengths when compared to cast UHTCC. Also for RC–UHTCC beams with an increase in the thickness of UHTCC layer, there was an increase in the stiffness, effectively gaining control over the cracks occurring in the concrete layer of the beam specimens [186]. FRP composites have been proven as a viable structural material in bridge construction. Bridge systems use FRP or hybrid FRP–concrete as primary construction materials for the application of bridge components such as girders, bridge decks, and slab-on-girder bridge systems. When compared to RC decks, hybrid FRP concrete decks reveals higher durability with less stiffness deterioration under design truckloads [187]. A fixed concrete bridge on the Indian River, Florida has been displayed in Figure 13b. Unprecedented threats from terrorist activities or natural disasters impose danger to public civil infrastructure such as bridges, and therefore, the impact and blast resistance design of such structures has become a prominent requirement in the design process. FRP material has been employed to strengthen and improve the impact resistance properties of the structures, including RC beams, RC slabs, RC columns, and masonry walls. This also results in an increase in the load-carrying capacities, ductility, energy absorption, and tensile strength of the materials with an increase in strain rate [188].

Deck panels: Flexure and shear strength seemed to be higher in all FRP composites when compared with RC for the application of bridge deck panels [189]. Decks made of hybrid fiber-reinforced composite materials were found to effectively fit for their design requirements. Glass and jute fibers reinforced with vinyl ester as matrix were used to fabricate a hybrid composite by hand lay-up technique [190].

Earthquake-resistant columns: FRP composites find an important application as a confining material for concrete in the construction of concrete-filled FRP tubes as earthquake-resistant columns and in the seismic retrofit of existing RC columns [191].

Pile material: Composite pile materials are the best replacement for traditional piles such as concrete, steel, and timber, as composite piles serve longer service life, require less maintenance costs, and are environmentally friendly. Hollow FRP piles show high potential in load-bearing applications and also provide significant advantages in terms of cost efficiency and structural capabilities [192,193].

Concrete slabs: For both unreinforced and RC slabs, carbon epoxy and E-glass epoxy composite systems restored original capacity of the damaged slabs, as well as resulted in a remarkable increase of more than 540% in the strength of the repaired slabs. Moreover, with the use of FRP systems, unreinforced specimens revealed a 500% improvement, while steel-reinforced specimens showed a 200% upgrade, in the structural capacity for retrofitting applications [194].

Sensors: Due to severe damages and collapses in civil structures, the need for development and advancement in sensing technology and sensors has given rise to structural health monitoring (SHM) technology. This consists of sensors, data acquisition, and transmission systems that can be used to monitor structural behavior and performance of structures when subjected to natural disasters such as an earthquake. The SHM system can record real loads, responses, and predict environmental actions [195,196].

### 4.2. Mechanical

Mechanical gear pair: For the application of gear pair, polyoxymethylene (POM) with 28% glass fiber reinforcement revealed significant enhancement of about 50% in the load-carrying capacity, with lower specific wear rate when compared to unreinforced POM [197]. Gear pair made of carbon–epoxy prepreg laminate was comparable to steel for the evaluation of static transmission error (STE) and mesh stiffness curves. Results showed a significant reduction in STE peak-to-peak value, which further resulted in improved noise, vibration, and harshness (NVH) performance of the material [198,199,200,201].

**Figure 14:**
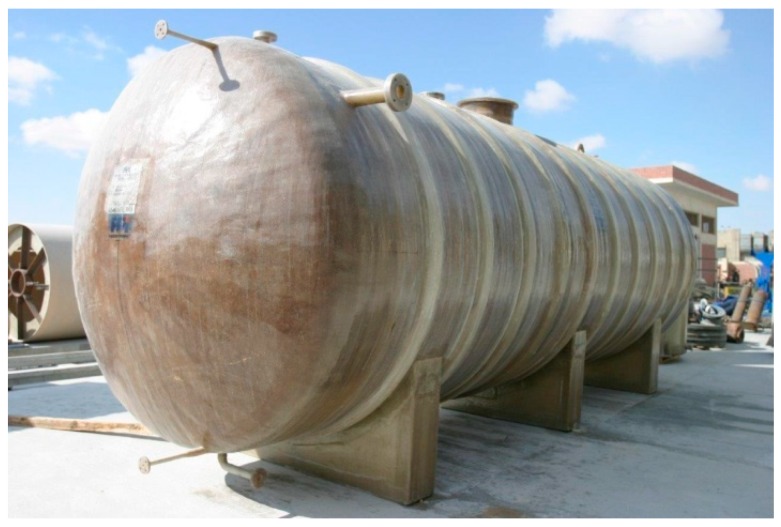
Pressure vessel made of thermosetting resin and fiberglass reinforcement, reproduced from [204] under open access license.

Pressure vessel: In the automobile industry, there is remarkable growth in the demand for lightweight material to increase fuel efficiency with a reduction of emission. FRP composites are serving these demands, for example, for safe and efficient storage and transportation of gaseous fuels such as hydrogen, and natural gas pressure vessels are used [129]. Pressure vessels made of FRP composite materials, when compared to metallic vessels, provide high strength and rigidity, improved corrosion resistance, and improved fatigue strength, besides being light in weight [202,203]. A pressure vessel made of thermosetting resin and fiberglass reinforcement is displayed in Figure 14.

Hydraulic cylinder: For the transportation of soil material, a dump truck uses a hydraulic system consisting of an actuator made of a telescopic hydraulic cylinder. There is a 96% weight reduction when the steel cylinder is replaced with a carbon fiber-reinforced epoxy resin composite. When this telescopic cylinder made of composite was installed, there was a 50% reduction in the whole hydraulic system [205].

Headstock material: During a typical machining operation, interference between machine tool and workpiece produces high vibration in the cutting tool relative to the workpiece. Nearly half of the deflection in cutting tools comes from the headstock; therefore, headstock demands a high degree of damping property. A hybrid steel–composite headstock adhesively manufactured by glass fiber epoxy composite laminates served a 12% increase in stiffness and 212% increase in damping property for the application of a precision grinding machine [206].

**Figure 15:**
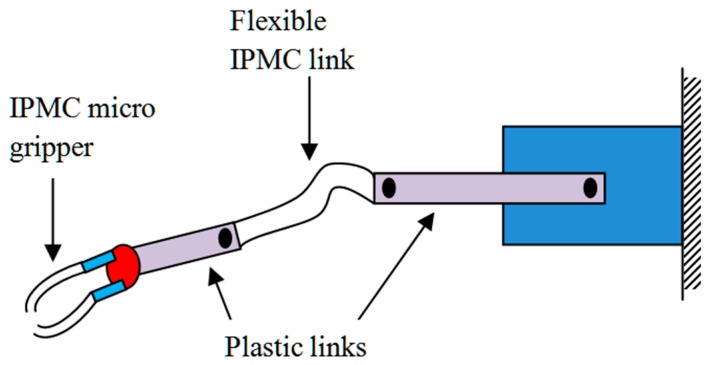
Flexible link manipulator.

Manipulator: A two-link flexible manipulator was developed using ionic polymer metal composite (IPMC), which manifests the potential of polymer-based composite materials for flexible joints and links in robotic assembly, as demonstrated in Figure 15. Sulfonated polyvinyl alcohol (SPVA), 1-ethyl-3-methylimidazolium tetrachloroaluminate (IL), and platinum (Pt) (SPVA/IL/Pt)-based IPMC manipulator links provide flexibility and compliant behavior during manipulating and handling of complex objects of different shapes and sizes [207].

Turbine blades: Turbine blades made of carbon fiber-reinforced silicon carbide (SiC) ceramic matrix composite (CMC) hold a bending strength of 350 MPa and fracture toughness of 4.49 $MPa\sqrt{m}$ when fiber content of 10–15% by volume is present [208].

### 4.3. Automobile

**Figure 16:**
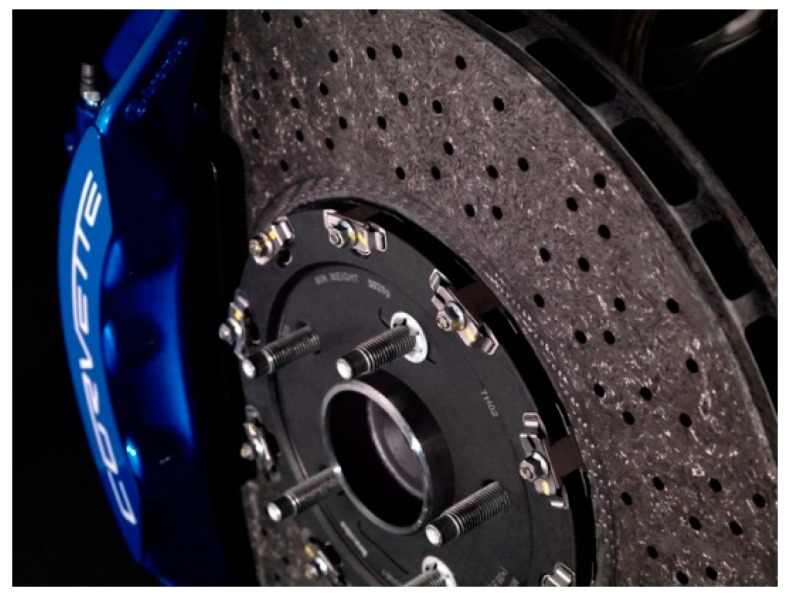
The braking system of corvette made of carbon–ceramic, which saved 4.9895 kg replacing iron, reproduced from [210] under open access license.

Braking system: In an automobile braking system, the temperature can reach up to thousands of degrees centigrade. A monolithic metal fails to perform well as they are not able to withstand these higher temperatures. Therefore, carbon fiber-reinforced silicon carbide (C-Si) finds applications in brake materials for heavy vehicles, high-speed trains, and emergency brakes in cranes [209]. Figure 16 shows a carbon–ceramic brake of a Chevrolet Corvette.

Trunk lid and body stiffener: In the transportation industry, CFRP fits as a reliable material for automobile body parts such as body stiffeners and engine hoods. As for this application, a higher strength to weight ratio is essential [211].

Bicycle: CFRP is replaced with hybridized carbon fiber with natural fibers, such as flax, to overcome the lower impact toughness and high cost of the material. A bicycle frame was fabricated using 70% flax fiber and 30% carbon fiber, which weighed just 2.1 kg and showed superior damping characteristics over aluminum, steel, and titanium [212].

**Figure 17:**
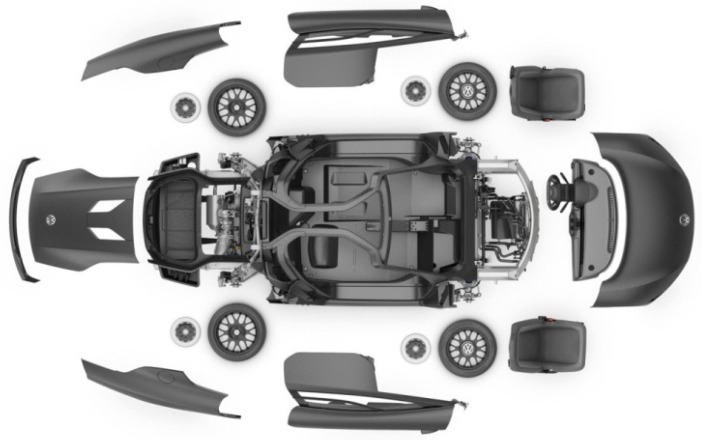
Volkswagen xl1 carbon fiber body parts, reproduced from [218] under open access license.

Automobile body parts: Automobile body parts, such as engine hood, dashboards, and storage tanks, are manufactured by using reinforcements of natural fibers such as flax, hemp, jute, sisal, and ramie. For these composite structures VARTM manufacturing method was employed and liability is tested with structural testing and by using impact stress analysis. The result showed a reduction in the weight of the material with the enhancement in stability and strength. The improvement in safety features were measured under head impact criteria (HIC), and it was found that composite structures comprised of natural fiber reinforcements are reasonable for automobile body parts [213,214,215,216,217]. Figure 17 displays exterior body parts of a model Volkswagen x11 made of carbon fiber.

Door panels: Addition of bamboo fibers increases the cell wall thickness of polyurethane composite structures, leading to the improvement of the sound absorption coefficient in automobile door panels [219,220].

Engine hood: Improvement in tensile strength and wear resistance properties have been observed for the engine hood material of an excavator engine when epoxy resin composite with reinforcement of glass fiber has been used over aluminum sheet metal [221].

**Figure 18:**
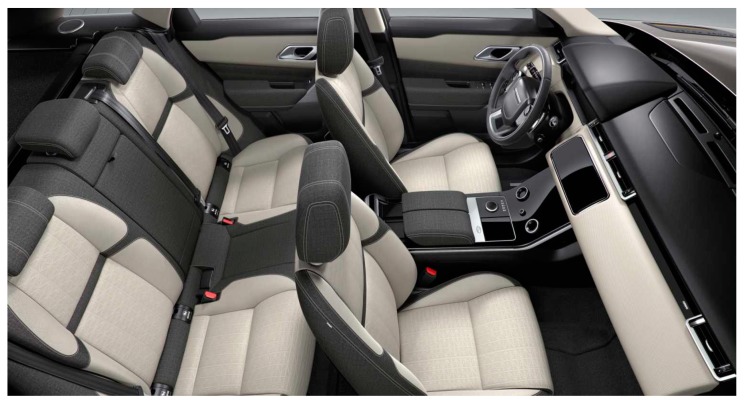
Car interior, reproduced from [224] under open access license.

Interior structures: The composite structure comprises of biodegradable natural fibers which have found significant applications as sound and vibration absorption material in interior automobile components. Composite laminate with bamboo, cotton, and flax fibers with PLA fibers showed bending stiffness of 2.5 GPa, which is higher than all other composites [222,223]. Figure 18 shows the interior structure of a car.

Engine frame: Steel engine subframe material, when replaced by carbon epoxy composite, displayed improvement in stiffness with a decrease in the maximum stress and weight from 16 kg to 5.5 kg [225].

T-joint: Epoxy resin composite with woven carbon fibers implemented for T-joint in the vehicle body revealed improvement in overall stiffness and strength behavior with a reduction in weight [226].

For the application of automobile bumper beam, glass/carbon mat thermoplastic (GCMT) composite has been designed and manufactured, which has shown improvement in impact performances with 33% weight reduction compared to the conventional glass mat thermoplastic (GMT) bumper beam [227].

### 4.4. Aerospace

Application of FRP composite materials in the field of aerospace industries can be seen with the implementation of highly durable, thermal-resistant, lightweight materials for the aircraft structure due to their outstanding mechanical, tribological, and electrical properties [69,74,228]. Natural fiber-reinforced thermoset and thermoplastic skins manifest the properties required for aircraft interior panels, such as resistance to heat and flame, serving easy recycling, and disposal of materials being cheaper and lightweight over conventional sandwich panels [229]. Though FRP composite shows a variety of applications in the aerospace industry due to their superior mechanical properties and lightweight structure, they face difficulty in recycling. To overcome this, natural fiber/biocomposite materials brought new prospects in the aerospace industry due to their biodegradability and lower cost [63].

Wireless signal transmission: Conductive fibers in the layer of fiber composite structure eliminate the requirement of separate wires for transceivers of communication devices. When voltage is applied to either layer of composite, it carries electric power to certain electric devices through the fibers [230].

The Hubble space telescope antenna: High stiffness with a lower coefficient of thermal expansion is achieved when P100 graphite fibers diffused in 6061 aluminum matrix composite material are employed to the high gain antenna of the Hubble space telescope [231].

Aircraft parts: Aircraft wing boxes made of ramie fiber composites revealed a 12–14% decrease in weight [232]. Hybrid kenaf/glass fiber-reinforced polymer composites showed enhanced mechanical properties with rain erosion resistance, suitable for aircraft application [233]. Carbon fiber-reinforced silicon carbide is applied for aircraft brakes to withstand temperatures up to 1200 °C [234].

Safety: The ablation method is carried out as one of the thermal protection methods for the spacecraft to ensure safety. An ablative composite material was used with zirconia fibers due to its significant mechanical properties and resistance to high-temperature ablation. It revealed that with 30% of zirconium fiber content composite material showed 19.33 MPa of bending strength; also at the higher temperature over 1400 °C, due to eutectic melting reaction, a ceramic protective layer forms which offers bending strength of 13.05 MPa [235].

### 4.5. Biomedical

Dentistry and orthopedic: Due to the strength characteristics and biocompatibility of fiber-reinforced composites, they are being used in the field of dentistry and orthopedics. Remarkable technological advances have been seen in the design of lower-limb sports prostheses [236]. For the reconstruction of craniofacial bone defects, new fiber-reinforced composite biomaterial replaces the material used for custom-made cranial implants [237]. A variety of aramid fibers display their biomedical applications in protein immobilization, for medical implants and devices, in modern orthopedic medicine, and as antimicrobial material. Typical polyamide (PA), i.e., nylon, is a synthetic polymer with high mechanical strength used in implants, and fibrous composites play a vital role in the manufacturing of dentures and suture materials. For antimicrobial applications, chitosan/m-aramid hybrids show enhancement in the surface area of assembled composites [238,239,240,241]. Biostable glass fibers reveal excellent load-bearing capacity in the implants, while antimicrobial properties are manifested by the dissolution of the bioactive glass particles that support bone-bonding [242].

Tissue engineering: Collagen–silk composite serves a promising application for reconstruction of lesioned tissues in tissue engineering. After fabricating the composite material by electrospinning, there is an increase in the ultimate tensile strength and elasticity of the material, with an increase in silk percentage [243]. Fibrous composite made of synthetic biodegradable polymers, polylactic co-glycolic acid (PLGA), gelatin, and elastin (PGE) scaffold can support dense cell growth and deliver tremendously high numbers of cells. This finds broad applicability in tissue engineering to meet design criteria necessary to generate scaffolds of natural and synthetic biomaterials [244,245]. A polyurethane cardiac patch loaded with nickel oxide (NiO) was fabricated using the electrospinning technique. When observed under SEM, PU/NiO nano-composite showed a reduction in the diameter of fibers and pores by 14% and 18%, respectively, compared to pure PU. Delayed blood clotting and a lower percentage of hemolytic revealed an improved antithrombogenic nature of PU/NiO nanocomposite, which plays a vital role in the repair of cardiac damage [246].

Wound healing: A fibrin–collagen filamentous polymer composite subjected to unconfined compression resulted in enhancement of elastic properties with increased node density and amalgamation between collagen and fibrin fibers. This led to the formation of a composite hydrogel, which further increased the modulus of shear storage at compressive strains. Fibrin has its active role in hemostasis and wound healing, while matrix gel based on collagen, gelatin, or elastin is utilized for scaffolds [247,248,249]. Biopolymers such as PLA, polyglycolic acid (PGA), PLGA, polycaprolactones (PCL), and polyesteramides (PEA) exhibit applications in biomedical fields to suture wounds, drug delivery, tissue engineering, fixing ligament/tendon/bone, dentistry, and surgical implants [250,251,252,253,254,255,256,257].

### 4.6. Marine

For marine applications, mechanical properties of materials get deteriorated in all types of metals, alloys, or composites due to seawater aging. Hybrid glass–carbon fiber-reinforced polymer composite (GCG_2_C)_s_ shows a high flexural strength of 462 MPa with the lowest water absorption tendency. Therefore retention of mechanical properties in hybrid (GCG_2_C)_s_ composite is more [258]. Moisture absorption properties exhibited by fiber composites are because of their structural or chemical composition, demonstrating various applications in the marine environment [259]. Due to the diffusion process, water molecules get absorbed in the material structure when it is exposed to the marine habitat. Diffusion in the material structure can be monitored by weight gain with respect to time. The number of water molecules that get absorbed is dependent upon the coefficient of diffusion of the material. Though the value of the coefficient of diffusion is lower in the composite materials, it is dependent upon various factors like the type of matrix material, the type of reinforcement material used, and the type of manufacturing process employed. Moisture absorption results in poor adhesion between the fiber and matrix in the composite structure, which ultimately deteriorates the properties of composite material [260,261,262,263,264,265].

Marine propeller: CFRP shows enhanced mechanical properties, such as high strength to weight ratio, resistance to corrosion, fatigue, and temperature changes with low cost of maintenance. These properties make CFRP a perfect fit for propeller material in marine applications [266].

Hull: Glass or carbon fiber skins with polymeric core sandwich composite panels have been used for the development of entire hulls and marine craft structures [267].

## 5. FRP Replacing Conventional Material

A variety of different fiber performances incorporated with composite materials, with the combination of distinct base materials and manufacturing techniques, offer an enhancement in properties of materials over pure metals, polymers, or alloys, which make FRP composites befitting for desired applications [268,269,270]. Composite materials with 5% MAPP by weight and 30% alkaline-treated hemp fibers by weight added to a PP matrix were found to be a replacement over pure PP, as an increment in flexural strength and tensile strength was found by 91% and 122%, respectively [78]. Flax/epoxy composite blades exhibit potential replacement characteristics, with respect to weight, structural safety, blade tip deflection, structural stability, and resonance, to replace glass/epoxy composite blades for small-scale horizontal axis wind turbine systems [271]. SEM morphology analysis revealed improvement in tensile and flexural strength due to good interface quality of RF/PP composite by 20.7% and 27.1%, respectively, when compared with pure PP [86]. A composite incorporated with PP and bamboo fiber reinforcements that were extracted by using an eco-friendly technique called solvent extraction, provided excellent fiber flexibility. The PP composite made of 20% bamboo fibers revealed the highest modulus of rupture (MOR), resulting in a rise in its flexural strength, which is an 8.3% increase to that of neat PP [272]. Conventional GMT was substituted by GCMT composite for the application of automobile bumper beams, which saw a 33% weight reduction with improved impact performances [227].

Fibers as reinforcement in a matrix of a composite structure act as a load-carrying element. While the matrix material keeps fibers in their required position and orientation, it also facilitates stress transfer and protection from the environment. FRP materials have been found to be superior to metals for a variety of applications where higher strength to weight ratio is required [273,274,275]. In recent years, polymer composites have shown a great potentiality and superiority over a prevalent yet critical issue of friction and wear faced by conventional metals and alloys [276,277,278]. Besides the remarkable tribological characteristics, polymeric composites offer flexibility in multifunctioning by tuning their composition to provide a cost-effective way of developing new tribological materials [279]. For automobile and aerospace applications, CF-MMC is replacing existing unreinforced metals and alloys as it provides excellent mechanical, thermal, and electrical properties with enhanced wear and corrosion resistance to withstand harsh environments [97]. The most common types of FRP used as reinforcement in the concrete structures are CFRP, GFRP, and aramid fiber-reinforced polymer (AFRP). These FRPs shows good resistance to shear and flexural stresses [280,281,282,283]. For the concrete structures to withstand in a harsh environment, reinforcement materials need to be noncorrosive and nonmagnetic. FRP bars possess these properties, which makes them applicable for the RC structures over the conventional steel reinforcement [284,285,286]. Structural material aluminum 6061 is replaced with hybridized flax and carbon fiber composites, as they revealed improvement in vibration damping properties in a material. A 252% gain in tensile strength with 141% improvement in damping ratio has been observed. In addition, there was a 49% weight saving due to a reduction in material density [149]. Hybridized composite structures with jute and carbon fiber reinforcements offer economic and sustainable alternatives over CFRP, revealing outstanding damping properties [148]. Engine hood material made of an aluminum sheet metal of an excavator engine was replaced with black epoxy composite with aluminum tri-hydroxide reinforced with glass fibers [151].

## 6. Challenges

A major challenge in fabricating FRC material is the lack of fiber–matrix characterization cognition. For the application of FRPCs in variety of fields, understanding their constituent’s significant material properties is necessary, with the basic constructs and the availability of manufacturing technology. For example, for the production of nanocomposites, one should acquire nanotechnology, including all the required tools and equipment. Also, the choice of manufacturing process eventually affects the final properties of material. Production volume influences the cost—the higher the volume of production, the less would be the cost of materials. Increasing production volume, in the case of the automobile industry, leads to greater risk of investing in raw materials while establishing manufacturing set-up according to the production rate and cycle time. Also the design complexity of the product augments the cycle time, slowing down the production rate.

Growing demand of high performance composites for aerospace and structural applications aggrandized the use of petroleum-based materials, leaving issue of composite waste disposal. However, nowadays, different researchers are developing various biocomposites using natural fibers and bio-based polymers, yet not all of these are completely biodegradable.

## 7. Conclusions

Composite materials are divulging numerous enhancements in distinct material properties since their invention in the last century. Copious amounts of research efforts have been made to discover optimized material to perform in a more effective way for desired applications. Over the past few decades, reinforcements of fibers or particles in the matrix structure of composite materials have revealed outstanding remarks, making them a popular choice for topmost applications.

Classifications of composite materials, along with the properties of their constituent elements, have been studied to understand the potentiality of different composite materials in various fields. Fiber-reinforced composite material was found to be one of the most promising and effective types of composites, as it claims dominance over the majority of applications from topmost fields.

There are numerous types of fibers available for fabrication of fiber-reinforced composites; those are categorized as natural and synthetic fibers. Synthetic fiber provide more stiffness, while natural fibers are cheap and biodegradable, making them environmentally friendly. Though both types of fibers have their efficacy in significant applications, latest research has revealed the exceptional performance of hybrid fiber-reinforced composite materials, as they gain the advantageous properties of both.

Composite materials are fabricated with a number of different techniques, among which every technique is applicable for certain material. Effectiveness of manufacturing technique is dependent on the combination of type and volume of matrix or fiber material used, as each material possesses different physical properties, such as melting point, stiffness, tensile strength, etc. Therefore, manufacturing techniques are defined as per the choice of material.

For distinct applications in a variety of fields, certain solitary materials might be replaced with composite materials, depending on the enhancement in its required property. Composite structures have shown improvement in strength and stiffness of material, while the reduction in weight is magnificent. Composites have also revealed some remarkable features such as resistance to impact, wear, corrosion, and chemicals, but these properties are dependent upon the composition of the material, type of fiber, and type of manufacturing technique employed to create it. In accordance with the properties required, composite materials find their applications in many desired fields.

More future research is intended to discover new composite structures with a combination of different variants and adopting new manufacturing techniques.

## Figures and Tables

**Table 1:** Matrix material used for some fibers with their applications and manufacturing techniques.

References	Materials Used		Application	Manufacturing Techniques
	Fiber Reinforcement	Matrix/Binder Material		
[64,65]	Carbon	PP, metals, ceramics, epoxy resin, Polyether ether ketone (PEEK)	Lightweight automotive products, fuel cells, satellite components, armor, sports.	Injection molding, filament winding, resin transfer molding (RTM)
[68]	Graphene	Polystyrene (PS), epoxy, Polyaniline (PANI)	Wind turbines, Gas tanks, aircraft/automotive parts.	CVD, pultrusion, hand/spray up method
[76]	Sisal	PP, PS, epoxy resin	Automobile body parts, roofing sheets	Hand lay-up, compression molding
[77]	Hemp	PE, PP, PU	Furniture, automotive.	RTM, compression molding
[80]	Kenaf	PLA, PP, epoxy resin	Tooling, bearings, automotive parts.	Compression molding, pultrusion
[83,84]	Flax	PP, polyester, epoxy	Structural, textile.	Compression molding RTM, spray/hand lay-up, vacuum infusion
[86,87]	Ramie	PP, Polyolefin, PLA	Bulletproof vests, socket prosthesis, civil.	Extrusion with injection molding
[89]	Rice Husk	PU, PE	Window/door frames, automotive structure.	Compression/injection molding
[92,93]	Jute	Polyester, PP	Ropes, roofing, door panels.	Hand lay-up, compression/ injection molding
[94,95]	Coir	PP, epoxy resin, PE	Automobile structural components, building boards, roofing sheets, insulation boards.	Extrusion, injection molding

**Table 2:** Some significant properties of frequently used fiber materials [114,115,116,117].

Fiber	Density (g/cm^3^)	Elongation (%)	Tensile Strength (MPa)	Young’s Modulus (GPa)
Aramid	1.4	3.3–3.7	3000–3150	63–67
E-glass	2.5	2.5–3	2000–3500	70
S-glass	2.5	2.8	4570	86
Cotton	1.5–1.6	3–10	287–597	5.5–12.6
Hemp	1.48	1.6	550–900	70
Jute	1.3–1.46	1.5–1.8	393–800	10–30
Flax	1.4–1.5	1.2–3.2	345–1500	27.6–80
Ramie	1.5	2–3.8	220–938	44–128
Sisal	1.33–1.5	2–14	400–700	9–38
Coir	1.2	15–30	175–220	4–6
Kenaf	0.6–1.5	1.6–4.3	223–1191	11–60
Bamboo	1.2–1.5	1.9–3.2	500–575	27–40
Oil palm	0.7–1.6	4–8	50–400	0.6–9
Betel nut	0.2–0.4	22–24	120–166	1.3–2.6
Sugarcane bagasse	1.1–1.6	6.3–7.9	170–350	5.1–6.2

**Table 3:** Variety of available matrix materials.

References	Matrix Material	Properties	Applications
[2]	Polyethersulfone	Flame resistant	Automotive
[3]	Polyphenylene sulfide	Resistance to chemicals and high temperature	Electrical
[3, 9]	Polysulfone	Low moisture absorption, high strength, low creep	Marine, food packaging
[6]	Polyethylene (PE)	Resistance to corrosion	Piping
[6,36,54,66, 94,96,101]	Polypropylene (PP)	Resistance to chemicals	Packaging, automotive, construction
[6,13,79]	Polylactic acid (PLA)	Biodegradable, non-toxic	Food handling, bio-medical
[10,90]	Polyurethane (PU)	Wear resistance, low cost, sound and water-proof	Structural, acoustic
[16]	Poly(butylene adipate-co-terephthalate)-PBAT	Biodegradable, high stiffness	Coating, packaging
[19]	Cement	Durable	Structural
[28]	Poly(vinyl alcohol	High tensile strength	Bio-medical
[33]	Natural rubber	Low density, low cost, biodegradable	Structural, automobile
[54,91,98,100, 102]	Epoxy resin	High strength	Automotive, aerospace, marine
[82,92]	Polyester	Durable, resistance to water, chemicals	Structural

## References

[1] Yashas Gowda T.G., Sanjay M.R., Subrahmanya Bhat K., Madhu P., Senthamaraikannan P., Yogesha B. (2018). SSPolymer matrix-natural fiber composites: An overview. Cogent. Eng..

[2] Sherif G., Chukov D., Tcherdyntsev V., Torokhov V. (2019). Effect of formation route on the mechanical properties of the polyethersulfone composites reinforced with glass fibers. Polymers.

[3] Chukov D., Nematulloev S., Zadorozhnyy M., Tcherdyntsev V., Stepashkin A., Zherebtsov D. (2019). Structure, mechanical and thermal properties of polyphenylene sulfide and polysulfone impregnated carbon fiber composites. Polymers.

[4] Linul E., Lell D., Movahedi N., Codrean C., Fiedler T. (2019). Compressive properties of Zinc Syntactic Foams at elevated temperatures. Compos. Part B Eng..

[5] Clyne T.W., Hull D. (2019). An Introduction to Composite Materials.

[6] Zagho M.M., Hussein E.A., Elzatahry A.A. (2018). Recent overviews in functional polymer composites for biomedical applications. Polymers.

[7] Monteiro S.N., de Assis F.S., Ferreira C.L., Simonassi N.T., Weber R.P., Oliveira M.S., Colorado H.A., Pereira A.C. (2018). Fique fabric: A promising reinforcement for polymer composites. Polymers.

[8] Movahedi N., Linul E. (2017). Quasi-static compressive behavior of the ex-situ aluminum-alloy foam-filled tubes under elevated temperature conditions. Mater. Lett..

[9] Chukov D., Nematulloev S., Torokhov V., Stepashkin A., Sherif G., Tcherdyntsev V. (2019). Effect of carbon fiber surface modification on their interfacial interaction with polysulfone. Results Phys..

[10] Linul E., Vălean C., Linul P.A. (2018). Compressive behavior of aluminum microfibers reinforced semi-rigid polyurethane foams. Polymers.

[11] Yongxu D., Dong L., Libin L., Guangjie G. (2018). Recent achievements of self-healing graphene/polymer composites. Polymers.

[12] Lebreton L.C.M., van der Zwet J., Damsteeg J.W., Slat B., Andrady A., Reisser J. (2017). River plastic emissions to the world’s oceans. Nat. Commun..

[13] Scaffaro R., Maio A., Lopresti F. (2018). Physical properties of green composites based on poly-lactic acid or Mater-Bi^®^ filled with Posidonia Oceanica leaves. Compos. Part. A Appl. S..

[14] Scaffaro R., Maio A. (2017). A green method to prepare nanosilica modified graphene oxide to inhibit nanoparticles re-aggregation during melt processing. Chem. Eng. J..

[15] Sun M., Sun X., Wang Z., Chang M., Li H. (2018). The influence of shape memory alloy volume fraction on the impact behavior of polymer composites. Polymers.

[16] Ferreira F.V., Cividanes L.S., Gouveia R.F., Lona L.M.F. (2017). An overview on properties and applications of poly (butylene adipate-co-terephthalate)-PBAT based composites. Polym. Eng. Sci..

[17] Dufresne A. (2019). Nanocellulose processing properties and potential applications. Curr. For. Rep..

[18] Habibi Y., Lucia L.A., Rojas O.J. (2010). Cellulose nanocrystals: Chemistry, self-assembly, and applications. Chem. Rev..

[19] Ferreira F., Pinheiro I., de Souza S., Mei L., Lona L. (2019). Polymer composites reinforced with natural fibers and nanocellulose in the automotive industry: A short review. J. Compos. Sci..

[20] Ardanuy M., Claramunt J., Toledo Filho R.D. (2015). Cellulosic fiber reinforced cement-based composites: A review of recent research. Constr. Build. Mater..

[21] Ardanuy M., Claramunt J., García-Hortal J.A., Barra M. (2011). Fiber-matrix interactions in cement mortar composites reinforced with cellulosic fibers. Cellulose.

[22] Balea A., Fuente E., Blanco A., Negro C. (2019). Nanocelluloses: Natural-based materials for fiber-reinforced cement composites. A critical review. Polymers.

[23] Golewski G.L. (2017). Determination of fracture toughness in concretes containing siliceous fly ash during mode III loading. Struct. Eng. Mech..

[24] Golewski G.L. (2017). Effect of fly ash addition on the fracture toughness of plain concrete at third model of fracture. J. Civ. Eng. Manag..

[25] Pickering K.L., Efendy M.G.A., Le T.M. (2016). A review of recent developments in natural fibre composites and their mechanical performance. Compos. Part. A-Appl. S..

[26] Alves Fidelis M.E., Pereira T.V.C., Gomes O.F.M., de Andrade Silva F., Toledo Filho R.D. (2013). The effect of fiber morphology on the tensile strength of natural fibers. J. Mater. Res. Technol..

[27] Lotfi A., Li H., Dao D.V., Prusty G. (2019). Natural fiber-reinforced composites: A review on material, manufacturing, and machinability. J. Compos..

[28] Pegoretti A., Fabbri E., Migliaresi C., Pilati F. (2004). Intraply and interply hybrid composites based on E-glass and poly (vinyl alcohol) woven fabrics: Tensile and impact properties. Polym. Int..

[29] Mehdikhani M., Gorbatikh L., Verpoest I., Lomov S.V. (2018). Voids in fiber-reinforced polymer composites: A review on their formation, characteristics, and effects on mechanical performance. J. Compos. Mater..

[30] Dickson A.N., Ross K.A., Dowling D.P. (2018). Additive manufacturing of woven carbon fibre polymer composites. Compos. Struct..

[31] Altenbach H., Altenbach J., Kissing W. (2004). Classification of Composite Materials. Mechanics of composite Structural Elements.

[32] Panthapulakkal S., Raghunanan L., Sain M., Birat K.C., Tjong J. (2017). Natural fiber and hybrid fiber thermoplastic composites. Green Compos..

[33] Nair A.B., Joseph R. (2014). Eco-friendly bio-composites using natural rubber (NR) matrices and natural fiber reinforcements. Chemistry, Manufacture and Applications of Natural Rubber.

[34] Agarwal B.D., Broutman L.J., Chandrashekhara K. (2017). Analysis and Performance of Fiber Composites.

[35] Dixit S., Goel R., Dubey A., Ahivhare P.R., Bhalavi T. (2017). Natural fibre reinforced polymer composite materials- A review. Polym. Renew. Resour..

[36] Arun Kumar D.T., Kaushik V.P., Raghavendra R.P.S. (2016). Tensile and impact properties of jute/glass and jute/carbon fiber reinforced polypropylene. J. Polym. Compos..

[37] Hempalaya (2019). The Difference between Hemp and Linen Fibers. https://hempalaya.com/blogs/news/der-unterschied-zwischen-hanf-und-leinen-fasern.

[38] Sunstrands (2019). The Basics of Kenaf Fiber and Hurd. https://www.sunstrands.com/2019/the-uses-of-kenaf-fiber/.

[39] Handloom Policies & Research (2017). Indira Gandhi Krishi Vishvavidyalaya (IGKV) Achieves a Breakthrough in Getting Linen Yarn Using the Flax Plant. https://www.unnatisilks.com/blog/indira-gandhi-krishi-vishvavidyalaya-igkv-achieves-a-breakthrough-in-getting-linen-yarn-using-the-flax-plant/.

[40] Textile School (2019). Sampling from Cotton Bales. https://www.textileschool.com/469/sampling-from-cotton-bales/.

[41] Lidija Grozdanic (2017). Students Use Rice Husks to Build Affordable Homes in the Philippines. https://inhabitat.com/students-use-rice-husks-to-built-affordable-homes-in-the-philippines/.

[42] (2019). Jute Geotextiles by Deyute, Geotextile Jute Fiber 732 gr/m^2^ 122 cm. https://www.deyute.com/product/geotextiles-natural-fibers/91.

[43] Backyard Poultry Contributor (2019). Chicken Feather & Skin Development. https://backyardpoultry.iamcountryside.com/chickens-101/chicken-feather-skin-development/.

[44] Tanmay Halaye (2019). Ramie Fiber Market Competitive Research and Precise Outlook 2019 to 2025. https://themarketresearchnews.com/2019/04/02/ramie-fiber-market-competitive-research-and-precise-outlook-2019-to-2025/.

[45] Textile Learner (2013). Abaca Fiber (Manila Hemp) Uses/Application of Abaca Fiber. https://textilelearner.blogspot.com/2013/04/abaca-fiber-manila-hemp-usesapplication.html.

[46] Fertilefibre Admin (2008). How We Make Our Peat-Free Coir Composts. https://www.fertilefibre.com/blog/peat-free-environment/coir-composts/.

[47] Pond (2017). Advantages of Using Natural Fibre Applications in Composites. https://pond.global/advantages-of-using-natural-fibre-applications-in-composites/.

[48] Black and Beautiful (2015). The Luffa/Loofah Skincare Benefits. https://blackandbeautiful.fr/blog/en/2015/04/08/the-luffa-skincare-benefits/.

[49] (2019). BabaMu, Sisal Fibers. https://pixabay.com/photos/sisal-sisal-fibers-sisal-palm-4319997/.

[50] Textile Learner (2014). Properties of Banana Fiber, Manufacturing Process of Banana Fiber, Application of Banana Fiber. https://textilelearner.blogspot.com/2014/01/properties-of-banana-fiber.html.

[51] Beyond Materials, Basalt Fiber. https://beyondmaterials.com.au/2019/03/16/basalt-fiber/.

[52] Carbon Black, Carbon. http://carbon-website.000webhostapp.com.

[53] Sanjay Impex Fiberglass Scree. http://sanjayimpex.com/fiber-glass.html.

[54] Sathishkumar T., Naveen J., Satheeshkumar S. (2014). Hybrid fiber reinforced polymer composites—A review. J. Reinf. Plast. Comp..

[55] Rahman R., Zhafer Firdaus S.P.S. (2019). Tensile properties of natural and synthetic fiber-reinforced polymer composites. Mechanical and Physical Testing of Biocomposites, Fibre-Reinforced Composites and Hybrid Composites.

[56] Jawaid M., Thariq M., Saba N. (2019). Mechanical and Physical Testing of Biocomposites, Fibre-Reinforced Composites and Hybrid Composites.

[57] Rajak D.K., Pagar D.D., Kumar R., Pruncu C. (2019). Recent progress of reinforcement materials: A comprehensive overview of composite materials. J. Mater. Res. Technol..

[58] Ghalia M.A., Abdelrasoul A. (2019). Compressive and fracture toughness of natural and synthetic fiber-reinforced polymer. Mechanical and Physical Testing of Biocomposites, Fibre-Reinforced Composites and Hybrid Composites.

[59] Abdellaoui H., Raji M., Essabir H., Bouhfid R., Qaiss A. (2019). Mechanical behavior of carbon/natural fiber-based hybrid composites. Mechanical and Physical Testing of Biocomposites, Fibre-Reinforced Composites and Hybrid Composites.

[60] Prakash S. (2019). Experimental investigation of surface defects in low-power CO_2_ laser engraving of glass fiber-reinforced polymer composite. Polym. Compos..

[61] Chalmers D.W. (1991). Experience in design and production of FRP marine structures. Mar. Struct..

[62] Unterweger C., Brüggemann O., Fürst C. (2013). Synthetic fibers and thermoplastic short-fiber-reinforced polymers: Properties and characterization. Polym. Compos..

[63] Yi X.S. (2015). Development of multifunctional composites for aerospace application. Multifunctionality of Polymer Composites.

[64] Haim A. (2017). Stability of composite stringer-stiffened panels. Stability and Vibrations of Thin Walled Composite Structures.

[65] Chung D.D.L. (2017). Introduction to carbon composites. Carbon Composites: Composites with Carbon Fibers, Nanofibers, and Nanotubes.

[66] Nobe R., Qiu J., Kudo M., Ito K., Kaneko M. (2019). Effects of SCF content, injection speed, and CF content on the morphology and tensile properties of microcellular injection-molded CF/PP composites. Polym. Eng. Sci..

[67] Xu Z., Gao C. (2015). Graphene fiber: A new trend in carbon fibers. Mater. Today.

[68] Sreenivasulu B., Ramji B., Nagaral M. (2018). A review on graphene reinforced polymer matrix composites. Mater. Today: Proc..

[69] Li Y., Wang S., Wang Q. (2017). A molecular dynamics simulation study on enhancement of mechanical and tribological properties of polymer composites by introduction of graphene. Carbon.

[70] Zhao X., Wang X., Wu Z., Keller T., Vassilopoulos A.P. (2018). Temperature effect on fatigue behavior of basalt fiber-reinforced polymer composites. Polym. Compos..

[71] Singh T.J., Samanta S. (2015). Characterization of Kevlar Fiber and its composites: A review. Mater. Today: Proc..

[72] Omrani E., Menezes P.L., Rohatgi P.K. (2016). State of the art on tribological behavior of polymer matrix composites reinforced with natural fibers in the green materials world. Eng. Sci. Technol. Int. J..

[73] Ouarhim W., Zari N., Bouhfid R., Qaiss A. (2019). Mechanical performance of natural fibers–based thermosetting composites. Mechanical and Physical Testing of Biocomposites, Fibre-Reinforced Composites and Hybrid Composites.

[74] Chand N., Fahim M. (2008). Sisal reinforced polymer composites. Tribol. Nat. Fiber Polym. Compos..

[75] Senthilkumar K., Saba N., Rajini N., Chandrasekar M., Jawaid M., Siengchin S., Alotman O.Y. (2018). Mechanical properties evaluation of sisal fibre reinforced polymer composites: A review. Constr. Build. Mater..

[76] Saxena M., Pappu A., Haque R., Sharma A. (2011). Sisal fiber based polymer composites and their applications. Cellulose Fibers: Bio-and Nano-Polymer Composites.

[77] Shahzad A. (2011). Hemp fiber and its composites—A review. J. Compos. Mater..

[78] Sullins T., Pillay S., Komus A., Ning H. (2017). Hemp fiber reinforced polypropylene composites: The effects of material treatments. Compos. Part. B-Eng..

[79] Ochi S. (2008). Mechanical properties of kenaf fibers and kenaf/PLA composites. Mech. Mater..

[80] Chin C.W., Yousif B.F. (2009). Potential of kenaf fibres as reinforcement for tribological applications. Wear.

[81] Abdi B., Azwan S., Abdullah M.R., Ayob A. (2014). Flexural and tensile behaviour of kenaf fibre composite materials. Mater. Res. Innov..

[82] Ben Mlik Y., Jaouadi M., Rezig S., Khoffi F., Slah M., Durand B. (2017). Kenaf fibre-reinforced polyester composites: Flexural characterization and statistical analysis. J. Text. Inst..

[83] Huang K., Tran L.Q.N., Kureemun U., Teo W.S., Lee H.P. (2019). Vibroacoustic behavior and noise control of flax fiber-reinforced polypropylene composites. J. Nat. Fibers.

[84] Goutianos S., Peijs T., Nystrom B., Skrifvars M. (2006). Development of flax fibre based textile reinforcements for composite applications. Appl. Compos. Mater..

[85] Habibi M., Laperrière L., Mahi Hassanabadi H. (2018). Replacing stitching and weaving in natural fiber reinforcement manufacturing, part 2: Mechanical behavior of flax fiber composite laminates. J. Nat. Fibers.

[86] Chen D., Pi C., Chen M., He L., Xia F., Peng S. (2019). Amplitude-dependent damping properties of ramie fiber-reinforced thermoplastic composites with varying fiber content. Polym. Compos..

[87] Du Y., Yan N., Kortschot M.T. (2015). The use of ramie fibers as reinforcements in composites. Biofiber Reinf. Compos. Mater..

[88] Majeed K., Arjmandi R., Al-Maadeed M.A., Hassan A., Ali Z., Khan A.U., Khurram M.S., Inuwa I.M., Khanam P.N. (2017). Structural properties of rice husk and its polymer matrix composites. Lignocellulosic Fibre and Biomass-Based Composite Materials.

[89] Arjmandi R., Hassan A., Majeed K., Zakaria Z. (2015). Rice husk filled polymer composites. Int. J. Polym. Sci..

[90] Wang Y., Wu H., Zhang C., Ren L., Yu H., Galland M.A., Ichchou M. (2018). Acoustic characteristics parameters of polyurethane/rice husk composites. Polym. Compos..

[91] Verma A., Negi P., Singh V.K. (2018). Experimental analysis on carbon residuum transformed epoxy resin: Chicken feather fiber hybrid composite. Polym. Compos..

[92] Das S., Singha A.K., Chaudhuri A., Ganguly P.K. (2019). Lengthwise jute fibre properties variation and its effect on jute–polyester composite. J. Text. Inst..

[93] Khan J.A., Khan M.A. (2015). The use of jute fibers as reinforcements in composites. Biofiber Reinforcements in Composite Materials.

[94] Munde Y.S., Ingle R.B., Siva I. (2018). Investigation to appraise the vibration and damping characteristics of coir fibre reinforced polypropylene composites. Adv. Mater. Process. Technol..

[95] Verma D., Shandilya A.K., Gupta A. (2013). Coir fibre reinforcement and application in polymer composites: A Review. J. Mater. Env. Sci..

[96] Chollakup R., Smitthipong W., Kongtud W., Tantatherdtam R. (2013). Polyethylene green composites reinforced with cellulose fibers (coir and palm fibers): Effect of fiber surface treatment and fiber content. J. Adhes. Sci. Technol..

[97] Liu Y., Ma Y., Yu J., Zhuang J., Wu S., Tong J. (2019). Development and characterization of alkali treated abaca fiber reinforced friction composites. Compos. Interface.

[98] Panneerdhass R., Gnanavelbabu A., Rajkumar K. (2014). Mechanical properties of luffa fiber and ground nut reinforced epoxy polymer hybrid composites. Proced. Eng..

[99] Bisen H.B., Hirwani C.K., Satankar R.K., Panda S.K., Mehar K., Patel B. (2018). Numerical study of frequency and deflection responses of natural fiber (Luffa) reinforced polymer composite and experimental validation. J. Nat. Fibers.

[100] Laban O., Mahdi E. (2016). Energy absorption capability of cotton fiber/epoxy composite square and rectangular tubes. J. Nat. Fibers.

[101] Panthapulakkal S., Sain M. (2006). Injection-molded short hemp fiber/glass fiber-reinforced polypropylene hybrid composites-Mechanical, water absorption and thermal properties. J. Appl. Polym. Sci..

[102] Hanan F., Jawaid M., Md Tahir P. (2018). Mechanical performance of oil palm/kenaf fiber-reinforced epoxy-based bilayer hybrid composites. J. Nat. Fibers.

[103] Ramesh M., Bhoopathi R., Deepa C., Sasikala G. (2018). Experimental investigation on morphological, physical and shear properties of hybrid composite laminates reinforced with flax and carbon fibers. J. Chin. Adv. Mater. Soc..

[104] Swolfs Y., Gorbatikh L., Verpoest I. (2014). Fibre hybridisation in polymer composites: A review. Compos. Part. A-Appl. S..

[105] Swolfs Y., Verpoest I., Gorbatikh L. (2018). Recent advances in fibre-hybrid composites: Materials selection, opportunities and applications. Int. Mater. Rev..

[106] Abhemanyu P.C., Prassanth E., Kumar T.N., Vidhyasagar R., Marimuthu K.P., Pramod R. (2019). Characterization of natural fiber reinforced polymer composites. AIP Conference Proceedings.

[107] Chawla N., Shen Y.L. (2001). Mechanical behavior of particle reinforced metal matrix composites. Adv. Eng. Mater..

[108] Mallick P. (2007). Fiber-Reinforced Composites: Materials, Manufacturing, and Design.

[109] Tanzi M.C., Farè S., Matthew D. (2019). Foundations of Biomaterials Engineering.

[110] Manickam G., Bharath A., Das A.N., Chandra A., Barua P. (2018). Thermoelastic stability behavior of curvilinear fiber-reinforced composite laminates with different boundary conditions. Polym. Compos..

[111] Fang K. (2019). Encapsulation Technologies for Electronic Applications.

[112] Aditya Narayana D., Ganapathia M., Pradyumna B. (2018). Investigation of thermo-elastic buckling of variable stiffness laminated composite shells using finite element approach based on higher-order theory. Compos. Struct..

[113] Kelly J., Mohammadi M. (2018). Uniaxial tensile behavior of sheet molded composite car hoods with different fibre contents under quasi-static strain rates. Mech. Res. Commun..

[114] Balakrishnan P., John M.J., Pothen L., Sreekala M.S., Thomas S. (2016). Natural fibre and polymer matrix composites and their applications in aerospace engineering. Advanced Composite Materials for Aerospace Engineering.

[115] Verma D., Senal I. (2007). Natural fiber-reinforced polymer composites. BiomassBiopolym.-Based Mater. Bioenergy.

[116] Kumar R., Ul Haq M.I., Raina A., Anand A. (2019). Industrial applications of natural fibre-reinforced polymer composites–challenges and opportunities. Int. J. Sustain. Eng..

[117] Menezes P.L., Rohatgi P.K., Lovell M.R. (2012). Studies on the tribological behavior of natural fiber reinforced polymer composite. Green Tribology.

[118] Venkatachalam N., Navaneethakrishnan P., Rajsekar R., Shankar S. (2016). Effect of pretreatment methods on properties of natural fiber composites: A review. Polym. Polym. Compos..

[119] Jamatia R., Deb A. (2017). Size effect in FRP-confined concrete under axial compression. J. Compos. Constr..

[120] Vincent T., Ozbakkaloglu T. (2013). Influence of fiber orientation and specimen end condition on axial compressive behavior of FRP-confined concrete. Constr. Build. Mater..

[121] Ozbakkaloglu T. (2013). Compressive behavior of concrete-filled FRP tube columns: Assessment of critical column parameters. Eng. Struct..

[122] Ozbakkaloglu T., Vincent T. (2014). Axial compressive behavior of circular high-strength concrete-filled frp tubes. J. Compos. Constr..

[123] Chattopadhyay S.K., Khandal R.K., Uppaluri R., Ghoshal A.K. (2010). Bamboo fiber reinforced polypropylene composites and their mechanical, thermal, and morphological properties. J. Appl. Polym. Sci..

[124] Zhao Y.Q., Zhou Y., Huang Z.M., Batra R.C. (2018). Experimental and micromechanical investigation of T300/7901 unidirectional composite strength. Polym. Compos..

[125] Chung D.D.L. (2017). Polymer-matrix composites: Structure and processing. Carbon Composites: Composites with Carbon Fibers, Nanofibers, and Nanotubes.

[126] Boisse P. (2015). Advances in Composites Manufacturing and Process Design.

[127] Balasubramanian K., Sultan M.T.H., Rajeswari N. (2018). Manufacturing techniques of composites for aerospace applications. Sustainable Composites for Aerospace Applications.

[128] Gascons M., Blanco N., Matthys K. (2012). Evolution of manufacturing processes for fiber-reinforced thermoset tanks, vessels, and silos: A review. Iie Trans..

[129] Holmes M. (2017). High volume composites for the automotive challenge. Reinf. Plast..

[130] Gunge A., Koppad P.G., Nagamadhu M., Kivade S., Murthy K.V.S. (2019). Study on mechanical properties of alkali treated plain woven banana fabric reinforced biodegradable composites. Compos. Commun..

[131] Elkington M., Bloom D., Ward C., Chatzimichali A., Potter K. (2015). Hand layup: Understanding the manual process. Adv. Manuf. Polym. Compos. Sci..

[132] Jamir M.R.M., Majid M.S.A., Khasri A. (2018). Natural lightweight hybrid composites for aircraft structural applications. Sustainable Composites for Aerospace Applications.

[133] Perna A.S., Viscusi A., Astarita A., Boccarusso L., Carrino L., Durante M., Sansone R. (2019). Manufacturing of a metal matrix composite coating on a polymer matrix composite through cold gas dynamic spray technique. J. Mater. Eng. Perform..

[134] Marques A.T. (2011). Fibrous materials reinforced composites production techniques. Fibrous and Composite Materials for Civil Engineering Applications.

[135] Ervina J., Ghaleb Z.A., Hamdan S., Mariatti M. (2018). Colloidal Stability of Water-based Carbon Nanotube Suspensions in Electrophoretic Deposition Process: Effect of Applied Voltage and Deposition Time. Compos. Part. A Appl. Sci..

[136] Carruthers J. (2018). Vacuum Bagging Process Overview, Coventive Composites. https://coventivecomposites.com/explainers/what-is-vacuum-bagging/.

[137] Awan F.S., Fakhar M.A., Khan L.A., Zaheer U., Khan A.F., Subhani T. (2018). Interfacial mechanical properties of carbon nanotube-deposited carbon fiber epoxy matrix hierarchical composites. Compos. Interface.

[138] Meola C., Boccardi S., Carlomagno G. (2017). Composite Materials in the aeronautical industry. Infrared Thermography in the Evaluation of Aerospace Composite Materials: Infrared Thermography to Composites.

[139] Ahmad N., Bilal I., Khattak S. (2018). Polyester usage in manufacturing of electrical and mechanical products and assemblies. Polyest.Prod. Charact. Innov. Appl..

[140] Davis D.C., Mensah T.O., Mensah T.O., Wang B., Bothun G., Winter J., Davis V. (2017). Fabrication and fatigue of fiber-reinforced polymer nanocomposites—a tool for quality control. Nanotechnology Commercialization: Manufacturing Processes and Products.

[141] Yalcinkaya M.A., Guloglu G.E., Pishvar M., Amirkhosravi M., Sozer M., Altan M.C. (2018). Pressurized Infusion (PI): A new and improved liquid composite molding process. J. Manuf. Sci. Eng..

[142] Plummer C.J.G., Bourban P.E., Månson J.A. (2016). Polymer matrix composites: Matrices and processing. Ref. Modul. Mater. Sci. Mater. Eng..

[143] Ishikawa H., Takagi H., Nakagaito A.N., Yasuzawa M., Genta H., Saito H. (2014). Effect of surface treatments on the mechanical properties of natural fiber textile composites made by VaRTM method. Compos. Interface.

[144] Mitschang P., Hildebrandt K. (2012). Polymer and composite moulding technologies for automotive applications. Advanced Materials in Automotive Engineering.

[145] Park C.H., Lee W.I. (2012). Compression molding in polymer matrix composites. Manufacturing Techniques for Polymer Matrix Composites (PMCs).

[146] Matveenko V.P., Kosheleva N.A., Shardakov I.N., Voronkov A.A. (2018). Temperature and strain registration by fibre-optic strain sensor in the polymer composite materials manufacturing. Int. J. Smart Nano Mater..

[147] Biswas B., Hazra B., Sarkar A., Bandyopadhyay N.R., Mitra B.C., Sinha A. (2018). Influence of ZrO^2^ incorporation on sisal fiber reinforced unsaturated polyester composites. Polym. Compos..

[148] Singh J.I.P., Singh S., Dhawan V. (2017). Effect of curing temperature on mechanical properties of natural fiber reinforced polymer composites. J. Nat. Fibers.

[149] Ramôa Correia J. (2013). Pultrusion of advanced fibre-reinforced polymer (FRP) composites. Advanced Fibre-Reinforced Polymer (FRP) Composites for Structural Applications.

[150] Verma D., Joshi G., Dabral R., Lakhera A. (2019). Processing and evaluation of mechanical properties of epoxy-filled E-glass fiber–fly ash hybrid composites. Mechanical and Physical Testing of Biocomposites, Fibre-Reinforced Composites and Hybrid Composites.

[151] Joshi S.C. (2012). The pultrusion process for polymer matrix composites. Manufacturing techniques for polymer matrix composites (PMCs).

[152] Leong Y.W., Thitithanasarn S., Yamada K., Hamada H. (2014). Compression and injection molding techniques for natural fiber composites. Natural Fibre Composites.

[153] Werner V.M.K., Krumpholz R., Rehekampff C., Scherzer T., Eblenkamp M. (2019). Thermoplastic encapsulations of a sensor platform by high-temperature injection molding up to 360 °C. Polym. Eng. Sci..

[154] González-López M.E., Pérez-Fonseca A.A., Manríquez-González R., Arellano M., Rodrigue D., Robledo-Ortíz J.R. (2018). Effect of surface treatment on the physical and mechanical properties of injection molded poly(lactic acid)-coir fiber biocomposites. Polym. Compos..

[155] Bhardwaj N., Kundu S.C. (2010). Electrospinning: A fascinating fiber fabrication technique. Biotechnol. Adv..

[156] Wang G., Yu D., Kelkar A.D., Zhang L. (2017). Electrospun nanofiber: Emerging reinforcing filler in polymer matrix composite materials. Prog. Polym. Sci..

[157] Gonzalez-Henriquez C.M., Sarabia-Vallejos M.A., Rodriguez Hernandez J. (2019). Polymers for additive manufacturing and 4D-printing: Materials, methodologies, and biomedical applications. Prog. Polym. Sci..

[158] Chua C.K., Leong K.F. (2017). 3D Printing and Additive Manufacturing: Principles and Applications. Fifth Edition of Rapid Prototyping.

[159] Goh G.D., Yap Y.L., Agarwala S., Yeong W.Y. (2018). Recent progress in additive manufacturing of fiber reinforced polymer composite. Adv. Mater. Technol..

[160] Hu C., Sun Z., Xiao Y., Qin Q. (2019). Recent patents in additive manufacturing of continuous fiber reinforced composites. Recent Pat. Mech. Eng..

[161] Parandoush P., Tucker L., Zhou C., Lin D. (2017). Laser assisted additive manufacturing of continuous fiber reinforced thermoplastic composites. Mater. Des..

[162] Shirvanimoghaddam K., Hamim S.U., Akbari M.K., Fakhrhoseini S.M., Khayyam H., Pakseresht A.H., Ghasali E., Zabet M., Munir K.S., Jia S. (2017). Carbon fiber reinforced metal matrix composites: Fabrication processes and properties. Compos. Part. A-Appl. Sci..

[163] Mantell S.C., Springer G.S. (1994). Filament winding process models. Compos. Struct..

[164] Minsch N., Herrmann F.H., Gereke T., Nocke A., Cherif C. (2017). Analysis of filament winding processes and potential equipment technologies. Procedure Cirp.

[165] Hopmann C., Wruck L., Schneider D., Fischer K. (2019). Automated winding of preforms directly from roving. Lightweight Des. Worldw..

[166] Sorrentino L., Anamateros E., Bellini C., Carrino L., Corcione G., Leone A., Paris G. (2019). Robotic filament winding: An innovative technology to manufacture complex shape structural parts. Compos. Struct..

[167] Mouritz A.P. (2012). Manufacturing of fibre–polymer composite materials. Introduction to Aerospace Materials.

[168] Frketic J., Dickens T., Ramakrishnan S. (2017). Automated manufacturing and processing of fiber-reinforced polymer (FRP) composites: An additive review of contemporary and modern techniques for advanced materials manufacturing. Addit. Manuf..

[169] Toutanji H., Deng Y. (2015). Comparison between Organic and Inorganic Matrices for RC Beams Strengthened with Carbon Fiber Sheets. J. Compos. Constr..

[170] Menna C., Asprone D., Ferone C., Colangelo F., Balsamo A., Prota A., Cioffi R., Manfredi G. (2013). Use of geopolymers for composite external reinforcement of RC members. Compos. Part. B-Eng..

[171] Trapko T. (2013). The effect of high temperature on the performance of CFRP and FRCM confined concrete elements. Compos. Part. B-Eng..

[172] Wang K., Young B., Smith S.T. (2011). Mechanical properties of pultruded carbon fibre-reinforced polymer (CFRP) plates at elevated temperatures. Eng. Struct..

[173] Ding Z., Dai J.G., Munir S. (2014). Study on an improved phosphate cement binder for the development of fiber-reinforced inorganic polymer composites. Polymers.

[174] Fang Y., Cui P., Ding Z., Zhu J.X. (2018). Properties of a magnesium phosphate cement based fire-retardant coating containing glass fiber or glass fiber powder. Constr. Build. Mater..

[175] Dai J.G., Munir S., Ding Z. (2014). Comparative study of different cement-based inorganic pastes towards the development of FRIP strengthening technology. J. Compos. Constr..

[176] Ding Z., Xu M.R., Dai J.G., Dong B.Q., Zhang M.J., Hong S.X., Xing F. (2019). Strengthening concrete using phosphate cement-based fiber-reinforced inorganic composites for improved fire resistance. Constr. Build. Mater..

[177] Manalo A., Aravinthan T., Fam A., Benmokrane B. (2017). State-of-the-Art Review on FRP Sandwich Systems for Lightweight Civil Infrastructure. J. Compos. Constr..

[178] Toutanji H.A., Gómez W. (1997). Durability characteristics of concrete beams externally bonded with FRP composite sheets. Cem. Concr. Comp..

[179] Kalfat R., Al-Mahaidi R., Smith S.T. (2013). Anchorage devices used to improve the performance of reinforced concrete beams retrofitted with frp composites: State-of-the-Art Review. J. Compos. Constr..

[180] Elgabbas F., Ahmed E.A., Benmokrane B. (2016). Flexural behavior of concrete beams reinforced with ribbed basalt-FRP bars under static loads. J. Compos. Constr..

[181] El Refai A., Abed F. (2015). Concrete contribution to shear strength of beams reinforced with basalt fiber-reinforced bars. J. Compos. Constr..

[182] Abed F., Alhafiz A.R. (2019). Effect of basalt fibers on the flexural behavior of concrete beams reinforced with BFRP bars. Compos. Struct..

[183] Zhang H.W., Smith S.T. (2012). Influence of FRP anchor fan configuration and dowel angle on anchoring FRP plates. Compos. Part. B-Eng..

[184] https://theconstructor.org/concrete/prestressed-concrete-principles-advantages/28/.

[185] (2018). Scullybob. https://en.wikipedia.org/wiki/17th_Street_Bridge_(Vero_Beach,_Florida).

[186] Huang B.T., Li Q.H., Xu S.L., Zhou B. (2019). Strengthening of reinforced concrete structure using sprayable fiber-reinforced cementitious composites with high ductility. Compos. Struct..

[187] Cheng L., Karbhari V.M. (2006). New bridge systems using FRP composites and concrete: A state-of-the-art review. Progr. Struct. Eng. Mater..

[188] Pham T.M., Hao H. (2016). Review of concrete structures strengthened with FRP against impact loading. Structures.

[189] Alagusundaramoorthy P., Harik I.E., Choo C.C. (2006). Structural behavior of FRP composite bridge deck panels. J. Bridge. Eng..

[190] Gopinath R., Poopathi R., Saravanakumar S.S. (2019). Characterization and structural performance of hybrid fiber-reinforced composite deck panels. Adv. Compos. Hybrid. Mater..

[191] Ozbakkaloglu T., Lim J.C., Vincent T. (2013). FRP-confined concrete in circular sections: Review and assessment of stress-strain models. Eng. Struct..

[192] Guades E., Aravinthan T., Islam M., Manalo A. (2012). A review on the driving performance of FRP composite piles. Compos. Struct..

[193] Sen R., Mullins G. (2007). Application of FRP composites for underwater piles repair. Compos. Part. B-Eng..

[194] Mosallam A.S., Mosalam K.M. (2003). Strengthening of two-way concrete slabs with FRP composite laminates. Constr Build. Mater..

[195] Ou J., Li H. (2010). Structural health monitoring in mainland China: Review and future trends. Struct. Health Monit. Int. J..

[196] Li H.N., Li D.S., Song G.B. (2004). Recent applications of fiber optic sensors to health monitoring in civil engineering. Eng. Struct..

[197] Mao K., Greenwood D., Ramakrishnan R., Goodship V., Shrouti C., Chetwynd D., Langlois P. (2019). The wear resistance improvement of fibre reinforced polymer composite gears. Wear.

[198] Catera P.G., Mundo D., Treviso A., Gagliardi F., Visrolia A. (2019). On the design and simulation of hybrid metal-composite gears. Springer Appl. Compos. Mater..

[199] Bae J.H., Jung K.C., Yoo S.H., Chang S.H., Kim M., Lim T. (2015). Design and fabrication of a metal composite hybrid wheel with a friction damping layer for enhancement of ride comfort. Compos. Struct..

[200] Shweiki S., Palermo A., Mundo D. (2017). A study on the dynamic behaviour of lightweight gears. Shock Vib..

[201] Rigaud E., Cornuault P.H., Bazin B., Grandais-Menant E. (2018). Numerical and experimental analysis of the vibroacoustic behavior of an electric window-lift gear motor. Arch. Appl. Mech..

[202] Schäkel M., Janssen H., Brecher C. (2019). Increased reliability for the manufacturing of composite pressure vessels. Lightweight Des. Worldw..

[203] Wilson A. (2017). Vehicle weight is the key driver for automotive composites. Reinf. Plast..

[204] Future Pipe Industries (2019). Tanks. https://www.futurepipe.com/products/tanks.

[205] Solazzi L., Buffoli A. (2019). Telescopic hydraulic cylinder made of composite material. Appl. Compos. Mater..

[206] Chang S.H., Kim P.J., Lee D.G., Choi J.K. (2001). Steel-composite hybrid headstock for high-precision grinding machines. Compos. Struct..

[207] Jain R.K., Khan A., Inamuddin I., Asiri A.M. (2018). Design and development of non-perfluorinated ionic polymer metal composite-based flexible link manipulator for robotics assembly. Polym. Compos..

[208] Lu Z.L., Lu F., Cao J.W., Li D.C. (2014). Manufacturing properties of turbine blades of carbon fiber-reinforced SiC Composite Based on Stereolithography. Mater. Manuf. Process..

[209] Patel M., Saurabh K., Prasad V.B., Subrahmanyam J. (2012). High temperature C/C–SiC composite by liquid silicon infiltration: A literature review. B. Mater. Sci..

[210] Stenquist P. Superbrakes for Civilians? The Cost Is the Obstacle 2010. https://www.nytimes.com/2010/08/01/automobiles/01BRAKES.html.

[211] Forintos N., Czigány T. (2019). Multifunctional application of carbon fiber reinforced polymer composites: Electrical properties of the reinforcing carbon fibers—A short review. Compos. Part. B Eng..

[212] Amiri A., Krosbakken T., Schoen W., Theisen D., Ulven C.A. (2017). Design and manufacturing of a hybrid flax/carbon fiber composite bicycle frame. Proc. Inst. Mech. Eng. Part. P J. Sports Eng. Technol..

[213] Kong C., Lee H., Park H. (2016). Design and manufacturing of automobile hood using natural composite structure. Compos. Part. B-Eng..

[214] Hassan S.M., Amir N., Rahmati A.M. (2014). Pedestrian safety investigation of the new inner structure of the hood to mitigate the impact injury of the head. Thin Wall. Struct..

[215] Koronis G., Silva A., Fontul M. (2013). Green composites: A review of adequate materials for automotive applications. Compos. Part. B-Eng..

[216] Kong C., Park H., Lee J. (2014). Study on structural design and analysis of flax natural fiber composite tank manufactured by vacuum assisted resin transfer molding. Mater. Lett..

[217] Alves C., Ferrão P.M.C., Silva A.J., Reis L.G., Freitas M., Rodrigues L.B. (2011). Ecodesign of automotive components making use of natural jute fiber composites. J. Clean. Prod..

[218] Belauto. https://belauto.com.my/2014-volkswagen-xl1-carbon-fiber-body-parts/.

[219] Ashworth S., Rongong J., Wilson P., Meredith J. (2016). Mechanical and damping properties of resin transfer moulded jute-carbon hybrid composites. Compos. Part. B-Eng..

[220] Flynn J., Amiri A., Ulven C. (2016). Hybridized carbon and flax fiber composites for tailored performance. Mater. Des..

[221] Wagh P.H., Pagar D.D. (2018). Investigation of mechanical and tribological behavior of composite material filled with black epoxy resin and aluminium tri-hydroxide using reinforcement of glass fiber. AIP Conf. Proc..

[222] Zhang J., Khatibi A.A., Castanet E., Baum T., Komeily-Nia Z., Vroman P., Wang X. (2019). Effect of natural fibre reinforcement on the sound and vibration damping properties of bio-composites compression moulded by nonwoven mats. Compos. Commun..

[223] Farid M., Purniawan A., Rasyida A., Ramadhani M., Komariyah S. (2017). Improvement of acoustical characteristics: Wideband bamboo based polymer composite. Iop Conf. Ser. Mater. Sci. Eng..

[224] Kelly J. (2017). What Is the Ideal Interior Material for Cars?. https://www.carconversation.com.au/opinions/what-is-the-ideal-interior-material-for-cars.

[225] Belingardi G., Koricho E.G. (2014). Design of a composite engine support sub-frame to achieve lightweight vehicles. Int. J. Automot. Compos..

[226] Hou W., Xu X., Han X., Wang H., Tong L. (2019). Multi-objective and multi-constraint design optimization for hat-shaped composite T-joints in automobiles. Thin Wall Struct..

[227] Kim D.-H., Kim H.-G., Kim H.-S. (2015). Design optimization and manufacture of hybrid glass/carbon fiber reinforced composite bumper beam for automobile vehicle. Compos. Struct..

[228] Barile C., Casavola C. (2019). Mechanical characterization of carbon fiber-reinforced plastic specimens for aerospace applications. Mechanical and Physical Testing of Biocomposites, Fibre-Reinforced Composites and Hybrid Composites.

[229] Alonso-Martin P.P., Gonzalez-Garcia A., Lapena-Rey N., Fita-Bravo S., Martinez-Sanz V., Marti-Ferrer F. (2012). Green Aircraft Interior Panels and Method of Fabrication. European Patent.

[230] Maryanka Y., Meidar M.I., Curless R.A. (2014). Method of Signal Transmission Using Fiber Composite Sandwich Panel. US Patent.

[231] Rawal S.P. (2001). Metal-matrix composites for space applications. JOM.

[232] Boegler O., Kling U., Empl D., Isikveren A. Potential of sustainable materials in wing structural design. Proceedings of the Deutscher Luft- und Raumfahrtkongress.

[233] Arockiam N.J., Jawaid M., Saba N. (2018). Sustainable bio composites for aircraft components. Sustainable Composites for Aerospace Applications.

[234] Fan S., Yang C., He L., Du Y., Krenkel W., Greil P., Travitzky N. (2016). Progress of ceramic matrix composites brake materials for aircraft application. Rev. Adv. Mater. Sci..

[235] Zou Z., Qin Y., Tian Q., Huang Z., Zhao Z. (2019). The influence of zirconia fibre on ablative composite materials. Plast. Rubber Compos..

[236] Scholz M.-S., Blanchfield J.P., Bloom L.D., Coburn B.H., Elkington M., Fuller J.D., Bond I.P. (2011). The use of composite materials in modern orthopaedic medicine and prosthetic devices: A review. Compos. Sci. Technol..

[237] Lazar M.A., Rotaru H., Bâldea I., Boşca A.B., Berce C.P., Prejmerean C., Câmpian R.S. (2016). Evaluation of the biocompatibility of new fiber-reinforced composite materials for craniofacial bone reconstruction. J. Craniofac Surg..

[238] Kowsari E., Haddadi-Asl V., Ajdari F.B., Hemmat J. (2019). Aramid fibers composites to innovative sustainable materials for biomedical applications. Materials for Biomedical Engineering.

[239] Teo A.J.T., Mishra A., Park I., Kim Y.-J., Park W.-T., Yoon Y.-J. (2016). Polymeric biomaterials for medical implants and devices. Acs Biomater. Sci. Eng..

[240] Kim S.S., Lee J. (2013). Antimicrobial polyacrylonitrile/m-aramid hybrid composite. Ind. Eng. Chem. Res..

[241] Kim S.S., Lee J. (2013). Miscibility and antimicrobial properties of m-aramid/chitosan hybrid composite. Ind. Eng. Chem. Res..

[242] Vallittu P.K., Närhi T.O., Hupa L. (2015). Fiber glass-bioactive glass composite for bone replacing and bone anchoring implants. Dent. Mater..

[243] Zhu B., Li W., Lewis R.V., Segre C.U., Wang R. (2014). E-Spun composite fibers of collagen and dragline silk protein: Fiber mechanics, biocompatibility, and application in stem cell differentiation. Biomacromolecules.

[244] Mengyan L., Mondrinos M.J., Xuesi C., Lelkes P.I. Electrospun blends of natural and synthetic polymers as scaffolds for tissue engineering. Proceedings of the 2005 IEEE Engineering in Medicine and Biology 27th Annual Conference.

[245] Lelkes P.I., Mengyan L., Mondrinos M., Ko F. U.S. Patent No. US8048446B2. https://patents.google.com/patent/US8048446B2/en.

[246] Jaganathan S.K., Mani M.P. (2018). Enriched mechanical, thermal, and blood compatibility of single stage electrospun polyurethane nickel oxide nanocomposite for cardiac tissue engineering. Polym. Compos..

[247] Kim O.V., Litvinov R.I., Chen J., Chen D.Z., Weisel J.W., Alber M.S. (2017). Compression-induced structural and mechanical changes of fibrin-collagen composites. Matrix Biol..

[248] Bensaıd W., Triffitt J., Blanchat C., Oudina K., Sedel L., Petite H. (2003). A biodegradable fibrin scaffold for mesenchymal stem cell transplantation. Biomaterials.

[249] Shevchenko R.V., James S.L., James S.E. (2010). A review of tissue-engineered skin bioconstructs available for skin reconstruction. J. R. Soc. Interface.

[250] Manvi P.K., Beckers M., Mohr B., Seide G., Gries T., Bunge C.-A. (2019). Polymer fiber-based biocomposites for medical sensing applications. Materials for Biomedical Engineering.

[251] Rebelo R., Fernandes M., Fangueiro R. (2017). Biopolymers in medical implants: A brief review. Procedure Eng..

[252] Azimi B., Nourpanah P., Rabiee M., Arbab S. (2014). Poly (lactide-*co*-glycolide) Fiber: An Overview. J. Eng. Fiber Fabr..

[253] Pivsa-Art W., Chaiyasat A., Pivsa-Art S., Yamane H., Ohara H. (2013). Preparation of polymer blends between Poly(Lactic Acid) and Poly(Butylene adipate-*co*-terephthalate) and biodegradable polymers as compatibilizers. Energy Proced..

[254] Shanks R., Kong I. (2012). Thermoplastic Elastomers. Applied Sciences.

[255] Panwiriyarat W., Tanrattanakul V., Pilard J.F., Pasetto P., Khaokong C. (2013). Preparation and Properties of Bio-based Polyurethane Containing Polycaprolactone and Natural Rubber. J. Polym. Env..

[256] Nicolae A., Grumezescu A.M. (2019). Polymer fibers in biomedical engineering. Mater. Biomed. Eng..

[257] Nandi S.K., Mahato A., Kundu B., Mukherjee P. (2019). Organic-inorganic micro/nanofiber composites for biomedical applications. Mater. Biomed. Eng..

[258] Jesthi D.K., Nayak R.K. (2019). Improvement of mechanical properties of hybrid composite through interply rearrangement of glass and carbon woven fabrics for marine applications. Compos. Part. B-Eng..

[259] Dhakal H.N., MacMullen J., Zhang Z.Y. (2016). Moisture measurement and effects on properties of marine composites. Marine Applications of Advanced Fibre-Reinforced Composites.

[260] Kootsookos A., Mouritz A.P. (2004). Seawater durability of glass- and carbon-polymer composites. Compos. Sci. Technol..

[261] Yu Y., Yang X., Wang L., Liu H. (2006). Hygrothermal ageing on pultruded carbon fibre/vinyl ester resin composite for sucker rod application. J. Reinf. Plast. Compos..

[262] Gellert E.P., Turley D.M. (1999). Seawater immersion ageing of glass-fibre reinforced polymer laminates for marine applications. Compos.A Appl. Sci. Manuf..

[263] Siriruk A., Jack Weitsman Y., Penumadu D. (2009). Polymeric foams and sandwich composites: Material properties, environmental effects, and shear-lag modelling. Compos. Sci. Technol..

[264] Siriruk A., Penumadu D., Weitsman Y. (2009). Effect of sea environment on interfacial delamination behaviour of polymeric sandwich structures. Compos. Sci. Technol..

[265] Akbar S., Zhang T. (2008). Moisture diffusion in carbon/epoxy composite and the effect of cyclic hygrothermal fluctuations: Characterization by dynamic mechanical analysis (DMA) and interlaminar shear strength (ILSS). J. Adhes..

[266] Kumar A., Lal K.G., Anantha S.V. (2019). Design and Analysis of a Carbon Composite Propeller for Podded Propulsion. Proceedings of the Fourth International Conference in Ocean. Engineering (ICOE2018), Lecture Notes in Civil. Engineering 22.

[267] Verma D., Goh K.L. (2019). Natural fiber-reinforced polymer composites. Biomass, Biopolymer-Based Materials, and Bioenergy: Construction, Biomedical, and Other Industrial Applications.

[268] Kovácik J., Jerz J., Mináriková N., Marsavina L., Linul E. (2016). Scaling of compression strength in disordered solids: Metallic foams. Frat. Ed Integrita Strutt..

[269] Movahedi N., Linul E., Marsavina L. (2018). The Temperature effect on the compressive behavior of closed-cell aluminum-alloy foams. J. Mater. Eng. Perform..

[270] Taherishargh M., Linul E., Broxtermann S., Fiedler T. (2018). The mechanical properties of expanded perlite-aluminium syntactic foam at elevated temperatures. J. Alloy. Compd..

[271] Park H. (2015). A study on structural design and analysis of small wind turbine blade with natural fibre (flax) composite. Adv. Compos. Mater..

[272] Tang Q., Wang Y., Ren Y., Zhang W., Guo W. (2019). A novel strategy for the extraction and preparation of bamboo fiber-reinforced polypropylene composites. Polym. Compos..

[273] Al-Mahaidi R., Kalfat R. (2018). Fiber-reinforced polymers and their use in structural rehabilitation. Rehabilitation of Concrete Structures with Fiber-Reinforced Polymer.

[274] Linul E., Marsavina L. (2011). Prediction of fracture toughness for open cell polyurethane foams by finite element micromechanical analysis. Iran. Polym. J..

[275] Linul E., Marsavina L. (2015). Assesment of sandwich beams with rigid polyurethane foam core using failure-mode maps. P. Rom. Acad. A.

[276] Rajak D.K., Mahajan N.N., Linul E. (2019). Crashworthiness performance and microstructural characteristics of foam-filled thin-walled tubes under diverse strain rate. J. Alloy. Compd..

[277] Marsavina L., Constantinescu D.M., Linul E., Voiconi T., Apostol D.A., Sadowski T. (2014). Evaluation of mixed mode fracture for PUR foams. Procedure Mater. Sci..

[278] Linul E., Serban D.A., Voiconi T., Marsavina L., Sadowski T. (2014). Energy-absorption and efficiency diagrams of rigid PUR foams. Key Eng. Mater..

[279] Pei X.Q., Friedrich K. (2016). Friction and wear of polymer composites. Reference Module in Materials Science and Materials Engineering.

[280] Habeeb M.N., Ashour A.F. (2008). Flexural behavior of continuous GFRP reinforced concrete beams. J. Compos. Constr..

[281] Abed F., El-Chabib H., AlHamaydeh M. (2012). Shear characteristics of GFRP-reinforced concrete deep beams without web reinforcement. J. Reinf. Plast. Compos..

[282] Rafi M.M., Nadjai A., Ali F. (2007). Experimental testing of concrete beams reinforced with carbon FRP. J. Compos. Mater..

[283] Rashid M.A., Mansur M.A., Paramasivam P. (2005). Behavior of aramid fiber-reinforced polymer reinforced high strength concrete beams under bending. J. Compos. Constr..

[284] Altalmas A., El Refai A., Abed F. (2015). Bond degradation of basalt fiber-reinforced polymer (BFRP) bars exposed to accelerated aging conditions. Constr. Build. Mater..

[285] Al-tamimi A., Abed F.H., Al-rahmani A. (2014). Effects of harsh environmental exposures on the bond capacity between concrete and GFRP reinforcing bars. Adv. Concr. Constr..

[286] El Refai A., Abed F., Altalmas A. (2014). Bond durability of basalt fiber-reinforced polymer bars embedded in concrete under direct pullout conditions. J. Compos. Constr..

